# FoCo: a simple and robust quantification algorithm of nuclear foci

**DOI:** 10.1186/s12859-015-0816-5

**Published:** 2015-11-21

**Authors:** Anastasiya Lapytsko, Gabriel Kollarovic, Lyubomira Ivanova, Maja Studencka, Jörg Schaber

**Affiliations:** 1 Medical Faculty, Institute for Experimental Internal Medicine, Otto von Guericke University, Pfälzer Platz 2, 39106 Magdeburg, Germany; 2 Cancer Research Institute, Slovak Academy of Sciences, Vlarska 7, 83391 Bratislava, Slovakia

**Keywords:** DNA double-strand break, γH2AX foci, Fluorescence microscopy, Image processing, Single cell analysis

## Abstract

**Background:**

The number of γH2AX foci per nucleus is an accepted measure of the number of DNA double-strand breaks in single cells. One of the experimental techniques for γH2AX detection in cultured cells is immunofluorescent labelling of γH2AX and nuclei followed by microscopy imaging and analysis.

**Results:**

In this study, we present the algorithm FoCo for reliable and robust automatic nuclear foci counting in single cell images. FoCo has the following advantages with respect to other software packages: i) the ability to reliably quantify even densely distributed foci, e.g., on images of cells subjected to radiation doses up to 10 Gy, ii) robustness of foci quantification in the sense of suppressing out-of-focus background signal, and iii) its simplicity. FoCo requires only 5 parameters that have to be adjusted by the user.

**Conclusions:**

FoCo is an open-source user-friendly software with GUI for individual foci counting, which is able to produce reliable and robust foci quantifications even for low signal/noise ratios and densely distributed foci.

**Electronic supplementary material:**

The online version of this article (doi:10.1186/s12859-015-0816-5) contains supplementary material, which is available to authorized users.

## Background

### γH2AX foci as DNA damage response measure

A variety of genotoxic stresses including ionizing radiation (IR) induce DNA damage [1]. DNA double-strand breaks (DSBs) are the most severe type of DNA damage, because their inaccurate repair can cause chromosomal translocations possibly leading to genomic instability and cancer development [1, 2].

For repairing DSBs cells utilize several repair pathways [1]. However, a key event prompting the DNA damage response is the phosphorylation of serine 139 of H2AX molecules, a histone H2A variant, on chromatin flanking DSB sites [3]. Phosphorylated H2AX (γH2AX) accumulate at DSBs sites creating a focus, which is required for the assembly of DNA damage repair proteins [1, 4–6]. γH2AX foci can be visualized in single cells using fluorescence microscopy (see below).

Recently, a direct correlation between the number of γH2AX foci and the number of DSBs was established [7, 8]. For mammalian cells the number of foci per cell increases with respect to DNA damage level roughly by a rate of 20–40 DSB foci per nucleus per Gy measured 30 min after irradiation [9]. Therefore, the quantification of γH2AX foci is widely used for estimating the number of DSBs and applied for modelling and understanding DNA damage repair processes in cells [10].

### Automatic methods for γH2AX foci quantification

A wide variety of experimental techniques has been developed for the detection of γH2AX foci in cultured cells [6, 11]. One of them is immunofluorescent labelling of γH2AX and nuclei followed by microscopy imaging and analysis [11]. The main advantage of this approach is the ability to provide quantitative information about the number of foci in single cells and, thus, the number of DSBs. However, this also requires sufficient image magnification and accurate image processing.

The conventional method of foci counting in microscopy images is manual counting. This is often criticized for being time-consuming and operator-biased [12–14]. Therefore, a range of both open access and commercial programs were developed for automatic foci detection [12–19]. The majority of these applications were created for processing high quality images and z-stacks obtained on high-end confocal laser-scanning microscopes [13, 15, 17–19]. Nevertheless, several studies were also dedicated to foci quantification on images obtained by wide-field fluorescent microscopes having a less well defined focal plane that usually causes an elevated background signal [14, 20]. These and other studies analyzed single cell images that were exposed to a radiation dose not exceeding 6 Gy [13–15, 17–20]. This is a radiation dose in which single foci can easily be identified both by eye and by automatic methods.

For our purposes, we needed to analyze images with nuclei and γH2AX foci for radiation doses up to 10 Gy. These images were characterized by i) dense and partially overlapping foci, especially 1–3 h after radiation, and ii) varying background both within and among images. Therefore, we tested various tools for automatic foci detection. In a literature search, the open source software tools CellProfiler [16], ImageJ [21], and FociCounter [14] were found to be the most promising tools for automatic foci counting [20]. However, FociCounter is a semi-automatic method, because it needs manual operations for nuclei detection. Therefore, we only used CellProfiler and ImageJ for foci quantification. We found various shortcomings in both of them, when applied to our images for time series of γ-irradiated cells for up to 10 Gy:

- Poor performance on images with densely distributed foci,
- Poor performance on images with low signal/noise ratio,
- Poor performance on images with varying background,
- Complicated to use.

In the following we use the term noise for both homogenous (background) as well as inhomogeneous noise.

### The aim of the study

To overcome above described limitations of existing software for automatic foci counting, we created a new algorithm. This is embedded in a new graphical user interface (GUI) FoCo, which was developed in Matlab together with ImageJ.

FoCo has the following key features exhibiting a unique combination of state-of-the-art image processing algorithms converging to a simple, yet robust method for automatic foci counting:

- *Reliability*: the ability to reliably quantify even densely distributed and/or overlapping foci, e.g., on images of cells subjected to radiation doses up to 10 Gy,
- *Robustness* of foci quantification in the sense of suppressing out-of-focus background signal,
- *Simplicity* of the algorithm: to analyze an image with FoCo the user has to provide only three parameters for nuclei identification and two parameters for foci identification.
- *Extendability*: users can modify the open source code of FoCo and implement further functionalities.

To validate foci quantifications in FoCo we created a test set of images obtained from a confocal laser-scanning microscope of MRC-5 normal human fibroblasts non-irradiated and γ-irradiated with a dose of 2.5 and 10 Gy. Then, we subjected the test set to manual counting by three operators setting the benchmark for a reliable foci count. We compared the automatic foci count in FoCo, CellProfiler and ImageJ to this benchmark. Foci quantifications in FoCo correlated best with three manual quantifications compared to CellProfiler and ImageJ (Additional file 1: Table S1, S2). In addition, we simulated a set of artificial foci images with a pre-defined number of foci, which we subjected to automatic foci quantifications. We compared the obtained quantification results with the reference foci numbers. Quantification results in FoCo deviated from reference less than 10 % confirming the reliability of quantifications.

To check the robustness of foci quantifications in FoCo we artificially blurred high quality images to different degrees and re-quantified foci using the three automatic methods. FoCo gave highly consistent results irrespective of image quality in contrast to CellProfiler and ImageJ, which were highly susceptible to changes in image quality. In addition, we utilized two sets of images of MRC-5 cells obtained from two independent γH2AX foci imaging analyses using confocal laser-scanning and conventional wide-field fluorescent microscopes, respectively. Each set contained images for several time points after cell irradiation. Despite different image qualities, FoCo showed almost indistinguishable results for both image sets.

## Experimental methods

### Cell culture and irradiation

MRC-5 human embryonic lung fibroblasts (ATCC, Cat. No. CCL-171™) at passage 8 (population doubling ~22) were cultured in Dulbecco’s modified Eagle’s medium (D-MEM) supplemented with 10 % foetal bovine serum (FBS)(Gibco), 100 units/ml MEM non-essential amino acids solution (Gibco) and 100 units/ml penicillin, 100 μg/ml streptomycin (Gibco). Cells were grown in a Thermo Scientific™ BBD 6220 CO2 incubator at 37 °C, 95 % humidity, 5 % CO_2_. DNA damage was induced by γ-irradiation: human primary fibroblast cells were exposed to ionizing radiation in a Biobeam GM 2000 (Gamma Medical Service) with ^137^Cs as radioactive isotope and a dose rate of approximately 3Gy/min.

### Immunofluorescent staining and image acquisition

The immunofluorescent staining of cell nuclei and γH2AX foci was performed according to the following protocol; cells grown on cover slips were washed in PBS and fixed in 4 % paraformaldehyde (in 1xPBS, pH 7.4) for 15 min at room temperature. After three washing steps with PBS, cells were permeabilized using 0.1 % Triton-X 100 (in 1xPBS, pH 7.4) for 15 min at room temperature and then incubated with the blocking reagent (5 % Bovine serum albumin in 1xPBS, pH 7.4) for 45 min. The primary antibody anti-γH2AX (Ab26350, Abcam) was diluted to 1:1000 in 1 % Bovine serum albumin, (in1xPBS pH 7.4) and added to the cells for 2 h at room temperature. After the incubation, cells on cover slips were washed three times in PBS and the fluorescent-labelled secondary antibody diluted 1:500 in the same buffer was added to cells (IgG-Alexa488, Cell Signaling #4408). The samples were stored in the dark at room temperature for 1 h. After washing, the DNA was stained with 49-6-diamidine-2-phenyl indole (DAPI, Invitrogen) diluted to a final concentration 1 μg/ml in the same buffer for 5 min at room temperature. Cells were then washed in PBS and mounted with the anti-fade medium (Vectashield).

Then cells were imaged using a confocal fluorescent laser scanning microscope (FluoView1000, Olympus) with a 60 × oil objective with numerical aperture (N.A.) equal to 1.35. In addition, cells were visualized using a conventional wide-field fluorescent microscope (Keyence BZ-8100E) with a 20× objective with N.A. equal to 0.4. As a result, TIF-images were obtained, where nuclei and foci were detected by blue and green channels, respectively.

## Implementation

### Used software

#### FoCo

For creating FoCo we used MatlabR2008b with Image Processing Toolbox (IPT) from http://www.mathworks.com [22]. In addition, we used ImageJ and MIJ that is a Java package for bi-directional communication and data exchange between Matlab and ImageJ. The package MIJ and the user documentation were obtained from the web page http://bigwww.epfl.ch/sage/soft/mij/. Thus, GUI FoCo combines the possibilities of two powerful image processing tools for foci counting.

The FoCo algorithm is described in detail in Additional file 1: sections S1, S2. The optimization of parameter values needed for the algorithm is described in Additional file 1: sections S3, S4. GUI FoCo with the source code is located in Additional file 2. Additional file 3 contains user documentation. Additionally, GUI FoCo is publicly available at https://sourceforge.net/projects/focicount along with the source code and user documentation. The software is distributed under GNU General Public License version 2.0 (GPL-2.0).

For foci counting in FoCo the user may utilize either RGB images that contain both nuclei and foci or grayscale images with nuclei and foci, respectively. In the case of RGB images it is assumed that nuclei and foci belong to different colour components of the image. Note that images must be in one of the following formats: TIF, JPEG, PNG or BMP.

**Fig. 1:**
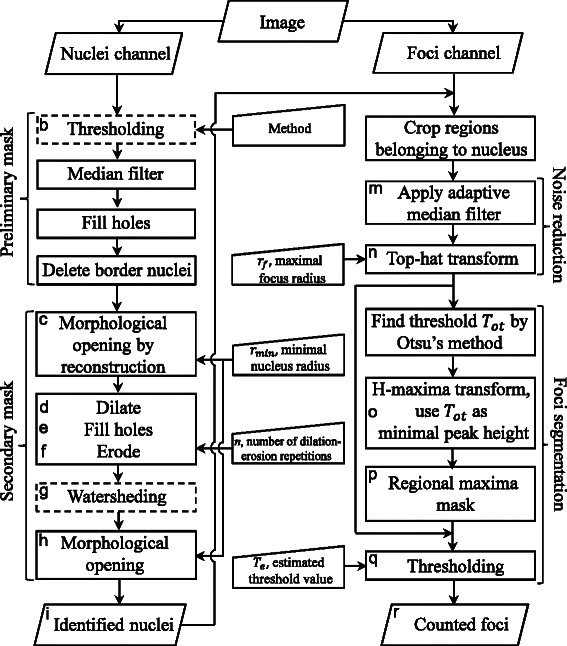
Flow chart with steps of the algorithm used to create FoCo. The left part depicts steps for nuclei identification. The right part depicts steps for foci identification. The middle part represents user-defined parameters of the algorithm. The majority of image processing steps were performed in Matlab. Procedures, which were performed in ImageJ, are designated by dashed frames. By grey letters we indicate steps that are visualized in Fig. 2 with corresponding images

**Fig. 2:**
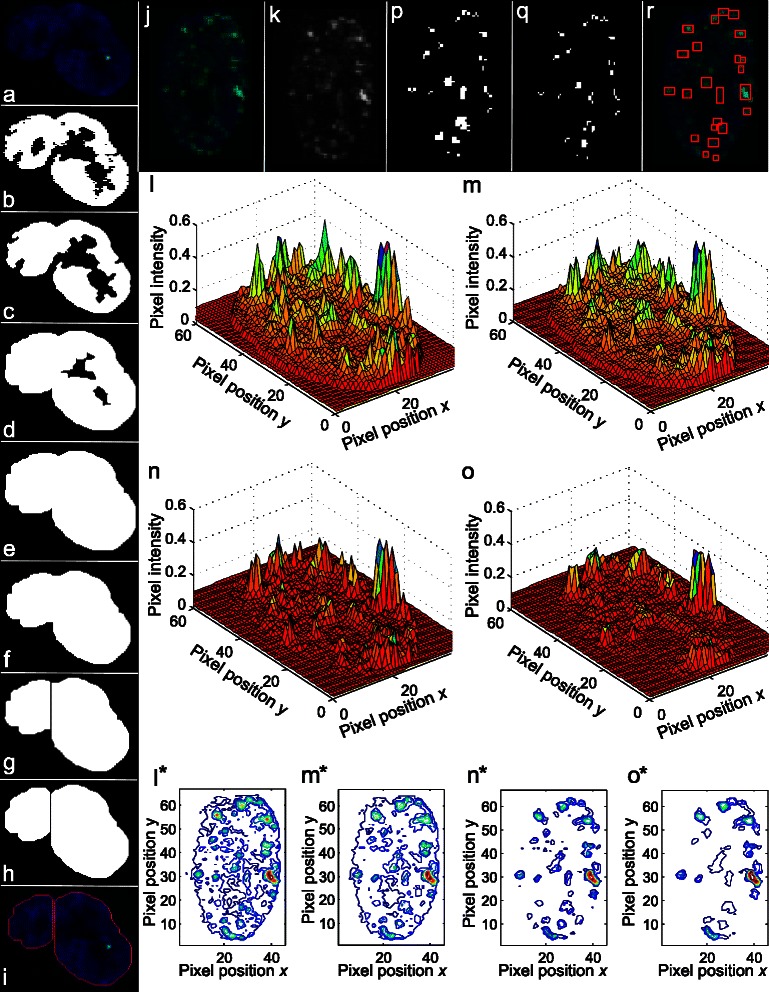
Visualization of main algorithm steps for nuclei and foci identification. The majority of image processing steps were performed in Matlab. The use of ImageJ is explicitly mentioned. **a** Nuclei image for demonstrating nuclei identification algorithm. **b** Thresholded blue component of the image (**a**) in ImageJ by Huang’s method. **c** Image (**b**) after filling holes, applying median filter of 3 × 3 size and morphological opening by reconstruction using a disk-shaped structuring element with radius 10. **d** Image (**c**) dilated by a 3 × 3 structuring element 3 times. **e** Image (**d**) with filled holes. **f** Image (**e**) eroded by the 3 × 3 structuring element 3 times. **g** Watersheding of the image (**f**) in ImageJ. **h** Morphological opening of the image (**g**) using a disk-shaped structuring element with radius 10. The result is a secondary mask. **i** Applying the secondary mask (**h**) to the image (**a**). **j** Image with the nucleus (blue) and foci (green) for demonstrating foci identification algorithm. **k** The green component of the image (**j**). **l** The 3D format of the image (**k**). Dimensions *x* and *y* indicate pixel positions in the intensity matrix of the foci image and dimension *z* indicates the pixel intensity value. Pixels belonging to foci have higher intensity than pixels belonging to the background and look like peaks. **m** Applying the adaptive median filter [22] to the image (**k**), (**l**). **n** Top-hat transformation of the image (**m**) using a disk-shaped structuring element with the radius *r*_*f*_ = 3. **o** H-maxima transformation of the image (**n**) using the Otsu’s threshold of the image (**n**) as a parameter. (**l** ^*^-**m** ^*^) Contour plots of images (**l**-**m**). **p** Regional maxima of the image (**o**). **q** Applying the mask (**p**) to the image (**n**) and thresholding with value *T*_*e*_ = 0.07. We designated obtained mask as a foci mask. Elements of the foci mask correspond to detected foci. **r** The original image (**j**) with identified foci marked by red frames

Figure 1 represents a flow chart with steps of the FoCo algorithm. Figure 2 visualizes some important steps of the algorithm with representative images. These steps are indicated by gray letters in Fig. 1 corresponding to image letters in Fig. 2. For a detailed description of the algorithm refer to the Additional file 1: section S1. Briefly, a two-channel immunofluorescence image (Fig. 2a, j) is split into the nuclei (blue in Fig. 2a, j) and foci (green in Fig. 2a, j) channels, respectively. The nuclear fraction is used to create a nuclear mask, by i) thresholding (Fig. 2b), ii) filling holes, median filtering and morphological opening by reconstruction (Fig. 2c). Then we apply iii) dilating (Fig. 2d), iv) filling holes (Fig. 2e) and v) eroding (Fig. 2f) to fill bay-regions inside the nuclei. Next we apply vi) watersheding to separate touching nuclei (Fig. 2g), and, vii) morphological opening to remove image elements that do not represent nuclei (Fig. 2h). This nuclear mask is applied to the original image to assign foci to specific nuclei (Fig. 2i). The foci per nucleus (Fig. 2k) are detected with respect to their intensities (Fig. 2l) creating a foci mask by applying i) the adaptive median filter for filtering out background noise (Fig. 2m), ii) the top-hat transform to correct for non-uniform illumination and remove the image background (Fig. 2n), and iii) applying the H-maxima transform to filter out non-relevant peaks (Fig. 2o), obtaining the foci mask (Fig. 2p). For the detailed description of the H-maxima transform refer to the Additional file 1: section S2. The foci-mask is again applied to the filtered foci image (Fig. 2n). The resulting image is thresholded to obtain the final countable foci (Fig. 2q). Finally, FoCo marks the obtained foci in the original image as a visual feedback to the user (Fig. 2r).

Taken together, FoCo employs a unique combination of techniques for noise reduction and object detection not used in earlier studies [12–15, 19, 20].

#### CellProfiler

CellProfiler 2.0 software installation package for Windows, user documentation and pipeline “Speckle Counting” were downloaded from the web page of CellProfiler www.cellprofiler.org. The optimization of parameter values needed for the algorithm is presented in the Additional file 1: section S5. The used pipeline with adjusted parameters is located in Additional file 4.

#### ImageJ

We downloaded ImageJ 1.45 s that is a public domain open source Java image processing program for Windows from http://imagej.nih.gov/ij/.

We used ImageJ as independent foci counting program as well as a part of FoCo. For foci quantification in ImageJ we basically followed the algorithm presented on the web page http://microscopy.duke.edu/HOWTO/countfoci.html. The customized macro with parameters and methods adapted for our images are available in the Additional file 1: section S6.

### Benchmarking FoCo, CellProfiler and ImageJ

To assess the performance of the automatic methods for foci counting, we compared each method to the results from three independent manual counts (Additional file 1: section S8). First, we conducted a weighted orthogonal regression and calculated the probability of the resulting correlation given the null hypothesis of a perfect 1:1-linear correlation. Second, we calculated a robust rank correlation coefficient (Additional file 1: section S9).

Additionally, we simulated artificial foci images containing a pre-defined number of foci and subjected them to automatic image analysis in FoCo, CellProfiler and ImageJ (Additional file 1: section S10). Then we compared obtained automatic quantification results with the pre-defined foci numbers.

## Results

To study the dynamics of the DNA damage response in MRC-5 primary human lung fibroblasts we performed γH2AX foci quantification on images obtained with a confocal laser scanning microscope (see Implementation Section). The image set contained pictures of non-irradiated cells at time points 1, 24, 72, 168 h after experiment start and images of cells after a dose of 2.5 and 10 Gy IR at time points 1, 3, 6, 24, 72, 168 h after irradiation. For each considered time point we analyzed from 3 to 9 images corresponding to about 100 cell nuclei. Refer to Additional file 5 for example images used in this study.

### Automatic foci quantifications

We automatically quantified foci using the freely available software CellProfiler [16] and ImageJ [21], which were found to be the most promising in a recent study [20]. We described the optimization of parameter values for CellProfiler and ImageJ in Additional file 1: sections S5, S6 and demonstrated foci detection on images in Additional file 1: Figure S8. An outlier analysis of obtained datasets revealed a presence of a few outliers, which were removed from all calculations presented in this section (Additional file 1: section S7).

**Fig. 3:**
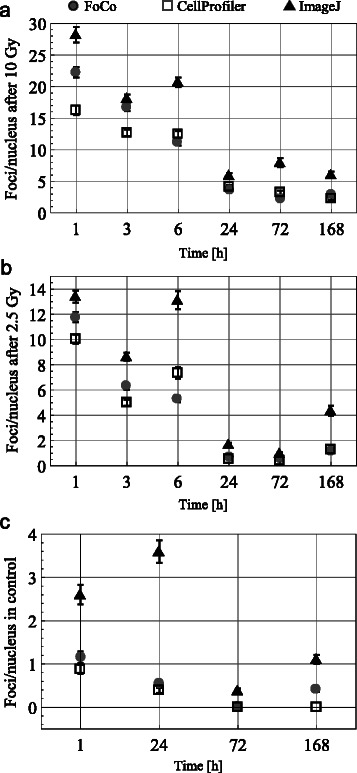
Results of automatic γH2AX foci quantification for images of MRC-5 cells from confocal laser-scanning microscope. Quantifications were performed in ImageJ, CellProfiler and FoCo. Image analysis of cells after 10 Gy irradiation (**a**) after 2.5 Gy irradiation (**b**) and control non-irradiated cells (**c**). Dots designate mean foci number per nucleus for considered time points after experiment start. Error bars designate standard error of the mean (SEM) (*n* ≥ 100)

Results of γH2AX foci quantification in CellProfiler and ImageJ are represented in Fig. 3a–c for cells after 10 Gy, 2.5 Gy IR and non-irradiated cells, respectively. According to quantifications with ImageJ the mean foci number per nucleus is clearly higher compared to quantifications with CellProfiler for all considered time points. In order to explain observed differences we analyzed foci counting algorithms in ImageJ and CellProfiler.

ImageJ defines foci as local maxima in the intensity matrix of the foci image corrected for a constant threshold. This is a simple method, which utilizes only one parameter for foci detection, i.e., the threshold value (‘Noise tolerance’). However, the algorithm does not take into account the variation of the background signal neither within the foci image nor between foci images. Therefore, this can lead to either overestimation or underestimation of foci numbers depending on the threshold value and the background intensity, which may change within or among images and nuclei (Additional file 1: Figure S8E).

In comparison, CellProfiler utilizes image processing modules for enhancing foci signal over background and subsequent thresholding of the foci image. However, thresholding alone without analysis of local or regional maxima may lead to clumping of potential foci and underestimation of foci number (Additional file 1: Figure S8D). In addition, we found it cumbersome to use CellProfiler, because of the many parameters the user has to adjust.

Thus, after comparing foci quantification results and analyzing foci counting algorithms in ImageJ and CellProfiler we decided to create our own foci quantification approach FoCo. FoCo aims to overcome limitations of CellProfiler and ImageJ such as i) poor performance on images with low signal/noise ratio; ii) poor performance on images with varying background; iii) difficulty to use.

We applied FoCo to count foci in images, which we used for foci quantification in CellProfiler and ImageJ. The optimization of FoCo parameter values is presented in the Additional file 1: section S3. In Additional file 1: Figures S7, S8C we demonstrated foci detection in FoCo on representative images. As for ImageJ and CellProfiler, foci quantification results in FoCo were subjected to outlier analysis (Additional file 1: section S7).

Figure 3 shows that quantification results in FoCo are located between quantification results of CellProfiler and ImageJ at time points 1 and 3 h for irradiated cells. For later time points quantification results from FoCo coincide or are located close to quantification results from CellProfiler. Additionally, quantification results from FoCo are located close to quantification results from CellProfiler for all time points for non-irradiated cells.

According to quantification results in FoCo the mean foci number per nucleus is monotonically decreasing in time 1 h after DNA damage for both 2.5 Gy and 10 Gy time series. This corresponds well to previous studies of γH2AX dynamics after irradiation [19, 23]. In contrast, quantifications in ImageJ show that the mean foci number per nucleus has a transient peak 6 h after both 2.5 and 10 Gy. Quantifications in CellProfiler demonstrate a transient plateau 6 h after 2.5 Gy. These non-consistent quantification for ImageJ and CellProfiler are probably because of changes in foci composition and increased background signal for the 6 h time point. Whereas CellProfiler underestimates earlier time points because of densely distributed foci and only starts to deliver reliable quantification after 3 h, ImageJ overestimates the 6 h quantification, because of the increased background signal.

### Benchmarking automatic foci quantifications

#### Manual quantifications

Performing automatic foci quantification by different methods we observed significant difference in quantification results. Therefore, we questioned how automatic foci quantifications would correlate with manual quantifications.

Manual foci count is time consuming and often criticized for being operator-biased. Nevertheless, manual foci count is still considered to be the gold standard and is regularly used as a benchmark to validate the performance of automatic methods [12–14]. Here, to minimize the operator bias and to define an objective benchmark, we considered manual foci counts from three independent operators to which the results of the automatic methods were compared (see Additional file 1: Figure S12 and sections S8, S9).

For manual foci counting we created a subset of 16 representative images. This test image set contained one image of control cells for each time point 1, 24, 72, 168 h and one or two images of cells post 2.5 Gy and 10 Gy IR for each time point 1, 3, 6, 24, 72, 168 h, respectively. Then the test image set was quantified manually by three independent operators and compared to the respective quantifications with FoCo, CellProfiler and ImageJ. Note that for the automatic quantification of the test image set we used the same parameters as for processing of the whole image set. The obtained quantification results were subjected to statistical analysis. Detailed results of automatic and manual foci quantifications for the test image set and details of statistical analysis are located in Additional file 1: sections S8, S9. According to the orthogonal regression and rank correlation analysis, foci quantifications with FoCo demonstrated better correlation with the three manual quantifications than ImageJ and CellProfiler (Additional file 1: Tables S1, S2).

#### Quantification of simulated images

Statistical analysis of manual and automatic foci quantifications favored FoCo over considered automatic methods. However, results of manual quantifications showed a high variability in quantification results at time points 1–6 h after 2.5 and 10 Gy IR. Shortly after irradiation foci are densely located and have a small size. This complicates distinguishing between foci and background and between foci located close to each other. This may result in observed variability in manual quantifications. For that reason, manual counting seems to be a poor benchmark for automatic analysis of our images at time points1-6 h after 2.5 and 10 Gy IR.

To this end, we decided to create an additional benchmark using artificial foci images with the pre-defined number of foci. Since all considered automatic approaches detected approximately the same number of cell nuclei (see Additional file 1: Table S3) and eventually average foci over the number of nuclei, we omitted simulation of nuclei images and focused on simulated foci images. We assumed that every simulated grayscale foci image corresponds to one nucleus.

**Fig. 4:**
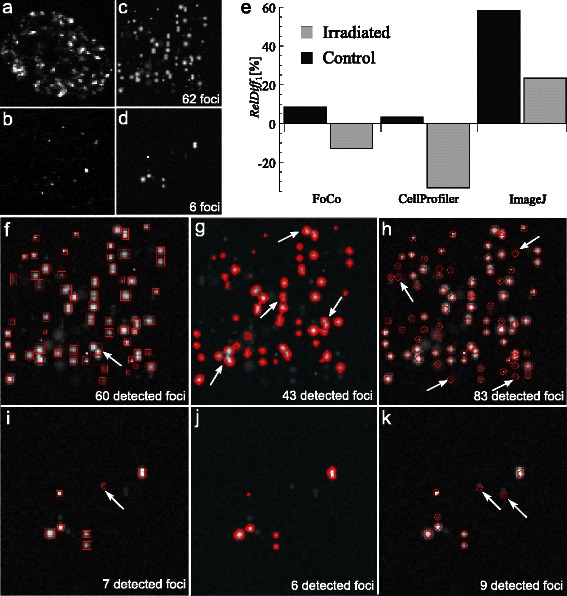
Simulation of artificial foci images with pre-defined number of foci. **a** Foci image of a cell 1 h after 10 Gy irradiation. **b** Foci image of a non-irradiated cell. **c** Representative simulated foci image with 62 foci (Irradiated in panel (**e**)). **d** Representative simulated foci image with 6 foci (Control in panel (**e**)). **e** Comparison between pre-defined foci numbers and automatic quantification results. The closer *RelDiff*_1_ to 0, the more precise are the quantification result obtained by the automatic method. **f**–**h** Demonstration of quantification results in FoCo, CellProfiler and ImageJ, respectively, applied to the representative image in panel C. **i**–**k** Demonstration of quantification results in FoCo, CellProfiler and ImageJ applied to the representative image in panel (**d**), respectively. Red frames, boundaries and circles designate detected foci in FoCo, CellProfiler and ImageJ, respectively. White arrows designate representative foci, which were either not split by the software or wrongly detected

For simulating artificial foci images we analyzed the foci and background structure of images obtained 1 h after 10 Gy IR and foci images of non-irradiated cells (representative foci images are depicted in Fig. 4a, b, respectively). As a result we created a range of focus templates representing a) single foci having different size and shapes (Additional file 1: Figure S14A-D, G, H) and b) two foci located close to each other (Additional file 1: Figure S14E, F). For simulating a single cell foci image we sampled foci templates, randomly put them on the empty image and added both homogeneous (background) and inhomogeneous noise. The program for simulating images saves the coordinates of placed foci and marks them by blue frames (Additional file 1: Figure S15A). This helps the user to distinguish between actual foci and background and perform benchmarking of automatic foci quantifications (Additional file 1: Figure S15B-D). In this section, blue frames are not visualized to avoid image overloading. For details of simulating foci images refer to the Additional file 1: section S10.

We simulated two image sets:

- “irradiated” - image set of 50 images mimicking foci images of irradiated cells and containing between 57and 69 foci per image. The representative image with 62 foci is depicted in Fig. 4c.
- “control” - image set of 50 images mimicking foci images of control cells and containing between 5 and 8 foci per image. The representative image with 6 foci is depicted in Fig. 4d.

Further, we subjected simulated images to the automatic analysis in FoCo, ImageJ and CellProfiler. Parameter values needed for automatic foci counting were optimized according to parameter optimization algorithms presented in Additional file 1: sections S3-S6. We compared obtained quantification results with pre-defined foci numbers using the relative difference *RelDiff*_1_:

$$ RelDif{f}_1=\frac{N_{Auto}-{N}_{Ref}}{N_{Ref}}\cdot 100\kern0.28em \% $$

Where *N*_*Ref*_ is a pre-defined reference mean foci number per image and *N*_*Auto*_ is a mean foci number per image obtained by an automatic method. A positive/negative value of *RelDiff*_1_ indicates an overestimation/ underestimation of the simulated foci number. The closer *RelDiff*_1_ to 0, the more precise are the quantification results obtained by the respective automatic method.

For FoCo *RelDiff*_1_ is about 10 % for the control image set and is about −10 % for the irradiated image set (Fig. 4e). Thus, FoCo slightly underestimates the foci number for the irradiated image set and slightly overestimates the foci number for the control image set (Fig. 4f, i). For CellProfiler *RelDiff*_1_ is close to 0 for the control image set and is about −33 % for the irradiated image set. Thus, CellProfiler demonstrates precise quantification results for the control image set, whereas it strongly underestimates quantification results for the irradiated image set (see Fig. 4g, j). ImageJ has *RelDiff*_1_ about 53 % and about 23 % for control and irradiated image sets, respectively. Thus, ImageJ strongly overestimates the number of simulated foci for both control and irradiated image sets (Fig. 4h, k).

To summarize, in comparison to CellProfiler and ImageJ, quantification results in FoCo vary from the reference value less than 10 % for both irradiated and control image sets indicating the reliability of quantifications. We provide the source code for simulating foci images (Additional file 6) for independent validation and as a basis for comparison of future algorithms. The CellProfiler pipeline, which was used for analyzing simulated images, can be found in Additional file 7. The representative simulated images depicted in Fig. 4c, d can be found in Additional file 8.

### Robustness analysis of automatic foci quantifications

To test the robustness of the automatic foci count methods with respect to signal/niose ratio, we artificially blurred high-quality images to different extend, re-quantified foci and compared results.

To this end, we selected two images of neighboring focal planes from a z-stack image of MRC-5 cells 1 h after 10 Gy irradiation obtained with a confocal laser-scanning microscope. We considered one of images as a base image. The second image we called the neighbor image. We used the neighbor image to create an artificial out-of-focus background signal for the base image, which would be similar to the background signal produced by wide-field fluorescent microscope. For that purpose, we split the neighbor image on channels and blurred the green component, which corresponds to foci signal. Blurring was performed in Matlab using circular averaging filter of radius *f* = 1, 2, 3, 4, 5 and 6 pixels: the higher the value of radius *f*, the higher blur-effect. Then, we added the obtained blurred green component of the neighbor image to the green component of the base image. In such way, we mimicked the effect of foci signal leaking from the neighboring focal plane into the base focal plane. The same procedure we applied to the z-stack image of MRC-5 cells 6 h after 10 Gy IR obtained on confocal laser-scanning microscope.

**Fig. 5:**
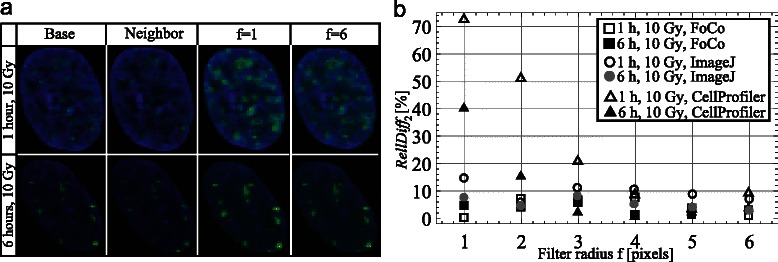
Analysis of images with artificial out-of-focus background signal. **a** Representative nuclei from base und neighbour images of cells 1 and 6 h after 10 Gy IR and corresponding base images with additional artificial out-of-focus background signal for two different strengths of blur-effect (*f* = 1, 6). **b** Relative differences *RelDiff*_*2*_ for images of cells 1 and 6 h after 10 Gy IR. Quantifications were performed in ImageJ, CellProfiler and FoCo. The lower *RelDiff*_*2*_, the more robust is the method in sense of suppressing out-of-focus background signal

Figure 5a demonstrates representative nuclei from base and neighbor images of cells 1 and 6 h after 10 Gy irradiation along with resulted images of nuclei with artificial out-of-focus background signal obtained for filter radius *f* = 1 and *f* = 6 pixels, respectively. Note that the larger the blur-effect, the less the neighbor signal influences the base signal, but the higher the background.

Afterwards, base images and images with the artificial out-of-focus background signal were subjected to the automatic foci count in FoCo, ImageJ and CellProfiler. Note that for processing of these images we used the same parameter values that we used for processing of the whole image set from the previous section.

Finally, as the measure of robustness, we quantified a relative difference *RelDiff*_2_:

$$ RelDif{f}_2=\frac{\left|{N}_{Base}-{N}_{Art}\right|}{N_{Base}}\cdot 100\%, $$

where *N*_*Base*_ and *N*_*Art*_ are mean foci numbers per nucleus on the base image and on the base image with the artificial out-of-focus background signal, respectively. Thus, the lower the relative difference *RelDiff*_2_, the more robust is the quantification method in sense of suppressing out-of-focus background signal.

As illustrated on Fig. 5b, *RelDiff*_2_ in FoCo varies maximally 7.5 % for both test images and all filter radii. In comparison, *RelDiff*_2_ in ImageJ is approximately 1.5 times higher than in FoCo in all instances. For CellProfiler *RelDiff*_2_ varies up to 73 %.

This analysis shows that in comparison with CellProfiler and ImageJ foci quantifications in FoCo are robust and insensitive to increased out-of-focus background signal. This implies that FoCo is able to produce reliable foci quantification results not only for images obtained on confocal laser-scanning fluorescent microscope, but also for images obtained on conventional wide-field fluorescent microscopes or, generally, on images with low signal/noise ratio.

### Validation of robustness of foci quantifications in FoCo

**Fig. 6:**
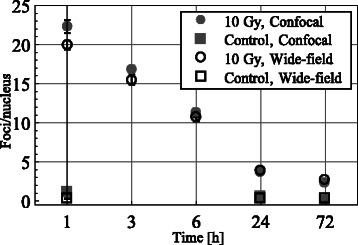
γH2AX foci quantifications in FoCo for images from confocal laser-scanning and wide-field fluorescent microscopes. Quantifications were performed for images of MRC-5 cells non-irradiated and after 10 Gy irradiation. Dots designate mean foci number per nucleus for considered time points after experiment start. Error bars designate standard error of the mean (SEM) (*n* ≥ 100)

To further explore the robustness of foci quantification with FoCo, we counted foci per nucleus on images of cells at 1, 3, 6, 24, and 72 h after 10 Gy IR that were obtained using both a wide-field and a confocal laser scanning microscope. Quantifications and parameter optimization were conducted as above using around 100 nuclei per time point (see Fig. 6).

Despite different image qualities (see Additional file 1: Figure S7, S8 and Figure S10) quantification results of both image sets do not substantially differ. One hour after 10 Gy irradiation the mean foci number per nucleus differs by 2.4 foci. For all other time points quantification differ less than 1.3 foci. The difference for control cells is less than 0.8 foci. Thus, FoCo delivers highly consistent foci counts for varying image qualities and signal/noise ratios.

## Discussion and conclusions

In this study, we presented the foci quantification algorithm FoCo, which was developed in Matlab together with ImageJ for counting individual γH2AX foci in microscopy images of single cells. Note that although FoCo has been tested for detection of γH2AX foci, it is a general approach, which can be applied for detection of all kinds of nuclear foci, e.g. 53BP1 foci [24].

It was earlier established that the initial amount of foci per nucleus is increasing with irradiation dose [3, 19]. This complicates the individual foci quantification for high irradiation doses. For the above mentioned reported value of 20–40 DSBs per Gy one can extrapolate around 300 foci per nucleus for 30 min after 10 Gy IR. We count 15–30 foci per nucleus 1 h after 10 Gy in one focal plane (Fig. 3) that are partially overlapping (Additional file 1: Figure S7, S8, S10). The main advantage of FoCo is the ability to perform reliable individual foci counting on images of cells subjected to doses up to 10 Gy IR. In comparison, previous studies on automatic foci count analyzed images of cells that were exposed to irradiation doses not exceeding 6 Gy [13–15, 17–20].

For verifying the reliability of automatic foci quantifications we applied a statistical analysis to compare automatic quantification results with manual quantification results (Additional file 1: sections S8, S9). As a result, foci quantifications in FoCo demonstrated better statistical correlation to manual quantifications than foci quantifications in CellProfiler and ImageJ. As an additional test for the reliability of automatic foci counting we simulated artificial foci images with a pre-defined number of foci and also subjected them to the automatic analysis in FoCo, CellProfiler and ImageJ. The comparison of obtained quantification results with reference foci numbers showed that quantification results in FoCo deviated from reference numbers less than 10 % for both low and high number of foci, which is the most stable and consistent result among the considered methods.

Another advantage of FoCo is the robustness of foci quantifications. FoCo proved to be insensitive to artificial out-of-focus background signal. We also compared foci quantifications of FoCo on images of MRC-5 cells obtained on a wide-field and a confocal laser scanning microscope, respectively. Despite the noticeable difference in image qualities, FoCo was able to deliver almost indistinguishable quantification results for both image sets. Thus, the demonstrated robustness of foci quantifications in FoCo is especially useful for images obtained with wide-field fluorescent microscopes. The robustness of quantification results in FoCo is achieved by applying several special techniques for i) noise reduction such as adaptive median filter [22], ii) object detection such as top-hat transform, and iii) robust maxima identification with H-maxima transform using Otsu’s threshold [22] as a parameter. This avoids both overestimation of the number of foci, because local maxima below a certain height are either disregarded or merged (Additional file 1: Figure S3).

However, apart from the quality of quantification results there are other factors, which play a role in choosing the appropriate software for foci counting:

- *availability*; FoCo, CellProfiler and ImageJ are freely available in the Internet. However, FoCo is a GUI in Matlab. Therefore, to run FoCo the user needs Matlab with the Image Processing Toolbox (IPT), which is a commercial software.
- *user-friendliness*; ImageJ has 3 parameters, FoCo has 5 parameters, CellProfiler has more than 10. The larger the amount of parameters, the more difficult and time consuming it is for the user to obtain an optimal parameter set. Here, the optimization of parameter for FoCo was implemented manually by the operator and did not require much effort. Here, we improved a previously proposed approach to find an optimal parameter set for image analysis [19].
- *batch processing*; FoCo and Cellprofiler are able to analyze a batch of images. For analyzing the batch of images in ImageJ a user-defined macro must be created.
- *visual feedback*; FoCo, CellProfiler and ImageJ are able to visualize both recognized nuclei and foci. However, FoCo and CellProfiler are able to relate numerical results of quantification to the visual representation of quantification.
- *extendability*; both FoCo and ImageJ can be extended by the user. Although CellProfiler cannot be extended by the user, it includes a lot of modules, which the user can add to the pipeline and apply if necessary.
- *processing time*; this includes not only the time needed to process images, but also to create a single table with quantification results. For processing the test image set FoCo needed 8 min and delivered results in a table format. CellProfiler needed 6 min for image processing. Then the operator spent 9 min to create a table with quantification results. ImageJ needed 17 min for image processing. The user of ImageJ spent 12 min to prepare data in one table format. In comparison, manual foci count of the test image set took about 3 h. Automatic foci quantifications were performed on PC with Intel(R) Core(TM) i7 CPU with 2.67 GHz and 8 GB RAM operating Windows 7.

The main features of FoCo, CellProfiler and ImageJ are summarized in Table 1.

**Table 1:** Comparison of FoCo, CellProfiler and ImageJ

Software	Availability	# of parameters	Batch analysis	Visual feedback	Processing time	Extendability
FoCo	Free GUI, but needs Matlab with IPT	5	Yes	Yes	8 min	Yes
CellProfiler	Free	>10	Yes	Yes	6 min + 9 min	No
ImageJ	Free	3	Yes	No	17 min +12 min	Yes

We conclude that FoCo is a user-friendly open source software for individual foci counting, which is able to produce reliable and robust foci quantifications even for low signal/noise ratios and densely distributed foci.

## Availability and requirements

**Project name:** FoCo

**Project home page:** https://sourceforge.net/projects/focicount

**Operating system(s):** tested under Windows

**Programming language:** Matlab

**Other requirements:** Image Pocessing Toolbox for Matlab, ImageJ, MIJ, Java Virtual Machine

**License:** GNU General Public License version 2.0 (GPL-2.0)

**Any restrictions to use by non-academics:** view license

## Additional files

Additional file 1: **Supplementary material for the main manuscript.** (S1) FoCo algorithm; (S2) H-maxima transform; (S3) Optimisation of parameter values *r*_*f*_ and *T*_*e*_: example with confocal microscope images; (S4) Optimisation of parameter values *r*_*f*_ and *T*_*e*_: example with wide-field microscope images; (S5) Optimisation of parameter values for CellProfiler: example with confocal microscope images; (S6) Optimisation of parameter values for ImageJ: example with confocal microscope images; (S7) Outlier detection; (S8) Manual foci quantifications; (S9) Comparison between automatic and manual foci quantifications; (S10) Simulation and analysis of foci images with pre-defined number of foci. (PDF 2638 kb)

Additional file 2: **GUI FoCo with source code.** (ZIP 69 kb)

Additional file 3: **Documentation for using FoCo.** (PDF 5946 kb)

Additional file 4: **CellProfiler pipeline for analyzing microscopy images.** (CP 9 kb)

Additional file 5: **Test image set.** The test image set contains RGB and grayscale microscopy images of MRC-5 cells 1 hour after 2.5 Gy and 3 hours after 10 Gy irradiation and of non-irradiated MRC-5 cells. The included image of MRC-5 cells after 10 Gy irradiation (‘10Gy.tif’) was used for creating documentation for using FoCo. The user may use this image to verify if he/she is using the program correctly. (ZIP 1961 kb)

Additional file 6: **Source code for simulating foci images and analyzing with FoCo algorithm.** The source code is written in Matlab. For simulating a foci image run the M-file ‘CreateAnalyzeArtificialImages.m’. (ZIP 5 kb)

Additional file 7: **CellProfiler pipeline for analyzing simulated images.** (CP 5 kb)

Additional file 8: **Simulated foci images.** We included simulated foci images from Fig. 4c–d used as representative images for demonstrating automatic foci quantifications. (ZIP 23 kb)

## Abbreviations

DSB: double-strand break
GUI: guided user interface
IPT: Image Processing Toolbox
IR: ionizing radiation
SEM: standard error of the mean
