# Supplementary material for: FoCo: a simple and robust quantification algorithm of nuclear foci
Source: BMC Bioinformatics. 2015 Nov 21;16:392. doi: 10.1186/s12859-015-0816-5 (PMC4654864; doi:10.1186/s12859-015-0816-5)
Supplement: Supplementary file 1 — Supplementary material for the main manuscript. (S1) FoCo algorithm; (S2) H-maxima transform; (S3) Optimisation of parameter values r f and T e: example with confocal microscope images; (S4) Optimisation of parameter values r f and T e: example with wide-field microscope images; (S5) Optimisation of parameter values for CellProfiler: example with confocal microscope images; (S6) Optimisation of parameter values for ImageJ: example with confocal microscope images; (S7) Outlier detection; (S8) Manual foci quantifications; (S9) Comparison between automatic and manual foci quantifications; (S10) Simulation and analysis of foci images with pre-defined number of foci. (PDF 2638 kb) [file 12859_2015_816_MOESM1_ESM.pdf]

# FoCo: a simple and robust quantification algorithm of nuclear foci

## Supplementary material

Anastasiya Lapytsko[1], Gabriel Kollarovic[1,2], Lyubomira Ivanova[1], Maja Studencka[1], Jörg Schaber[1*]

[1] Institute for Experimental Internal Medicine, Medical Faculty, Otto von Guericke University, Pfälzer Platz 2, 39106, Magdeburg, Germany

[2] Cancer Research Institute, Slovak Academy of Sciences, Vlarska 7, 83391, Bratislava, Slovakia

[*] To whom correspondence should be addressed:

Jörg Schaber
Phone: +49 391 67 50227
Fax: +49 391 67 13312
Email: schaber@med.ovgu.de

**Table of Contents**

S1. FoCo algorithm ..... 2

S2. H-maxima transform ..... 5

S3. Optimization of parameter values $r_f$ and $T_e$: example with confocal microscope images ..... 6

S4. Optimization of parameter values $r_f$ and $T_e$: example with wide-field microscope images ..... 11

S5. Optimization of parameter values for CellProfiler: example with confocal microscope images ..... 12

S6. Optimization of parameter values for ImageJ: example with confocal microscope images ..... 12

S7. Outlier detection ..... 14

S8. Manual foci quantifications ..... 14

S9. Comparison between automatic and manual foci quantifications ..... 15

S10. Simulation and analysis of foci images with pre-defined number of foci ..... 20

References: ..... 22

# S1. FoCo algorithm

### Nuclei identification

The algorithm for nuclei identification is represented in the left part of the flow chart in Figure 1. All steps of the algorithm are performed one by one applying the next operation to the result of the previous one. We demonstrate steps of the algorithm using a nuclei image with two touching nuclei from Figure 2A, where the nuclei are visualized in the blue channel by DNA staining.

For the nuclei identification we segment nuclei from the nuclei image creating a nuclear mask. The nuclear mask is a black-and-white bitmask of the same size as the original image with white pixels, where nuclei are identified, and black pixels elsewhere [1]. We divided the procedure of creating the nuclear mask on two parts: i) creating a preliminary mask, and ii) creating a secondary mask. This is achieved by applying basic and advanced procedures of segmentation, noise and morphological image processing, respectively.

We start the algorithm with creating a preliminary mask. In the first step, we apply a global thresholding that is a standard procedure for image segmentation [2]. To this end, the nuclei image is transferred from FoCo to ImageJ and an appropriate thresholding method is chosen depending on image quality. The '*thresholding method'* is the first user-defined parameter. In our studies, we used Huang's method for processing image sets obtained on both confocal laser-scanning  and wide-field fluorescent microscopes.

Thresholding alone is often not enough to get satisfactory segmentation results (see Figure 2B). Therefore, we combined thresholding in ImageJ with additional image processing manipulations in Matlab and ImageJ.

After processing in ImageJ we transfer the thresholded image back to FoCo and suppress "salt&pepper" noise applying an adaptive median filter with default size of 3×3 pixels.

Further, we fill holes in the nuclei. Holes may appear after thresholding due to, e.g., inhomogeneous DNA staining.

Then we exclude nuclei that touch the image border to keep only entirely visible nuclei.

In this way, we designate obtained image as a preliminary mask (see Figure 2C). For some images, the preliminary mask may provide a satisfactory nuclei segmentation. However, usually the preliminary mask needs additional processing steps that we perform creating a secondary mask.

First, we apply a morphological opening by reconstruction [2] to the preliminary mask. The aim of this process is to remove image elements that do not represent nuclei (dust elements, etc.) and that were not suppressed by the median filter. Morphological opening by reconstruction implements following operations:

1. eroding the preliminary mask using a disk-shaped structuring element with radius $r_{\min}$ (in pixels) removing image elements that fit into the structuring element.
2. restoring exactly the shapes of image objects that remain after erosion.

The radius $r_{\min}$ is the second user-defined parameter of the algorithm and designates the '*minimum radius of the nucleus*'. In our studies, we used $r_{\min} = 10$ pixels.

Second, we fill bay-regions inside the nuclei that are not yet recognized as nuclei, because they are not completely enclosed by nuclear regions. For this we apply following procedures:

1. dilate the image using a 3×3 structuring element *n* times (see Figure 2D),
2. fill holes (see Figure 2E),
3. erode the image using the same 3×3 structuring element *n* times (see Figure 2F).

The '*number of dilation-erosion repetitions' n* is the third user-defined parameter of the algorithm. In our studies, we used *n*=3.

In order to split touching nuclei we transfer the image from FoCo to ImageJ, apply the watershed transform (see Figure 2G) and transfer the image back to FoCo.

Finally, we apply morphological opening operation with the disk-shaped structuring element of radius $r_{\min}$ that was used for morphological opening by reconstruction. For the binary image, the morphological opening is a union of all translations of the structuring element that fit entirely within the image [2]. Therefore, morphological opening suppresses regions of identified nuclei that cannot contain the structuring element, smoothes nuclei contours, breaks thin connections and removes thin protrusions [2].

As a result, we created the secondary mask (see Figure 2H,I) and segmented nuclei from the background.

**Foci identification**

The algorithm for foci identification was implemented completely in Matlab. The steps of the algorithm are presented in the right part of the flow chart in Figure 1. In the first step, we apply the nuclei mask to the foci image. Then we crop automatically regions of the foci image that belong to identified nuclei and process them one by one.

We demonstrate remaining steps of the algorithm using a cropped nucleus with foci shown in Figure 2J. The green component of the image (Figure 2K) is the corresponding foci image. For convenience, we also represent the foci image in three-dimensional format with a corresponding contour plot (see Figure 2L). Dimensions *x* and *y* indicate pixel positions in the intensity matrix of the foci image and dimension *z* indicates the intensity value of the pixel. Pixels that belong to foci have higher intensity than pixels that belong to the background and look like peaks (Figure 2L).

The aim of the next step of the algorithm is to reduce image noise that may originate from imaging or staining processes, i.e., "salt&pepper" noise. For this we apply an adaptive median filter to the foci image using a fixed size of 3×3 pixels. The M-file that implements adaptive median filter was taken from Gonzalez et al. [2]. Figure 2M demonstrates the result of applying the adaptive median filter to the foci image from Figure 2L.

In the next step we correct a possible non-uniform illumination of the image and remove the image background. For this we apply to the image a top-hat transform [2], which includes following operations:

1. morphological image opening. For grayscale images, the opening first erodes the image using a disk-shaped structuring element with the radius $r_f$ in pixels. Then the morphological opening dilates the eroded image by the same element. As the result, the image opening removes elements smaller than the structuring element, i.e. foci, and produces a reasonable estimate of the background across the image [2].
2. subtracting the morphologically opened image, i.e. estimated background, from the original one and making the background of the image even.

Thus, the radius of the structuring element $r_f$ designates the '*maximum radius of foci*' and serves as the fourth user-defined parameter of the algorithm. For the image from Figure 2M we used $r_f = 3$. The transformed image is illustrated in Figure 2N. In Figure S1A,B we demonstrated processes of erosion and subsequent dilation by the disk-shaped structuring element with the radius $r_f = 3$ applied to the foci image from Figure 2M. We recommend an optimization procedure for the parameter $r_f$ in Supplementary Section S3.

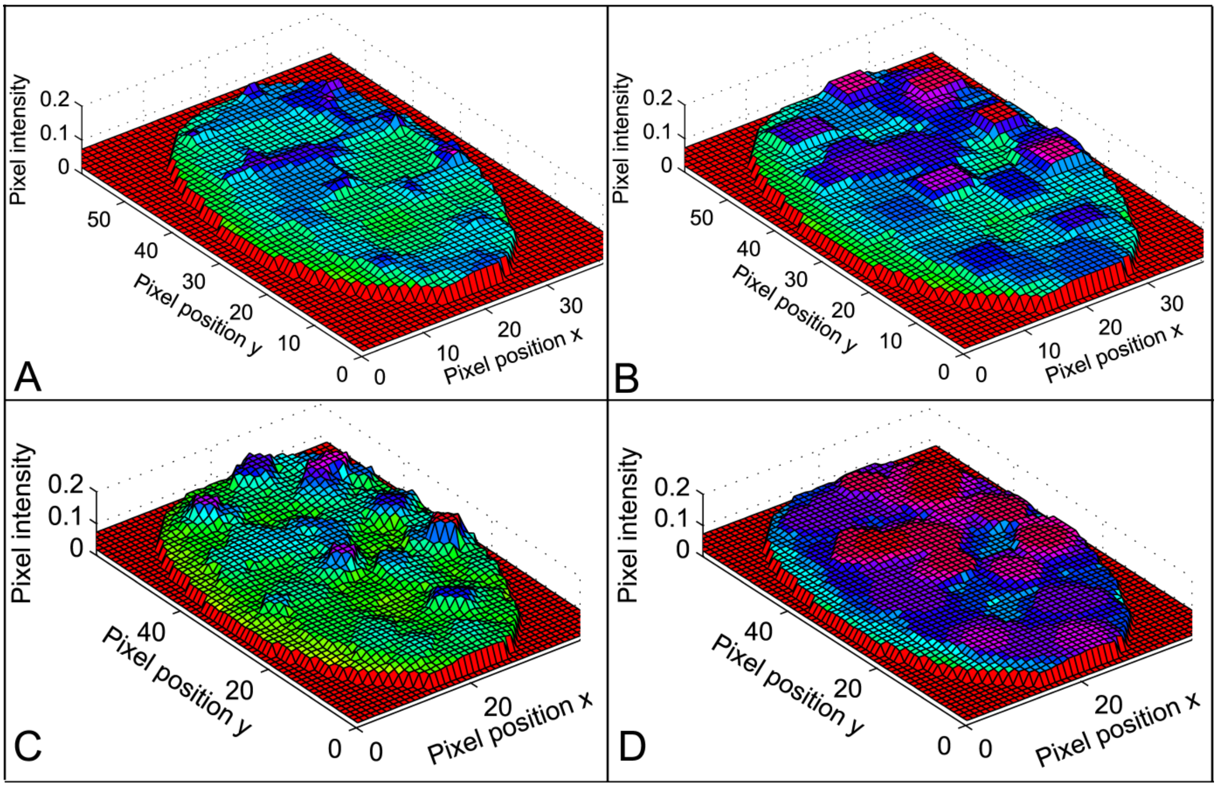


**Figure S1**. **Visualization of the morphological opening of the foci image from Figure 2M. (A)** Erosion of the image with the structuring element disk of radius $r_f = 3$. **(B)** Dilation of the image from Panel A with the same structuring element. **(C)** Morphological opening with the structuring element disk of radius $r_f = 2$. **(D)** Morphological opening with the structuring element disk of radius $r_f = 4$.

Afterwards, we perform the image segmentation to separate foci from the background. Shortly, after irradiation (< 3 hours) foci are distributed with high density in the nucleus [3]. Therefore, in comparison with nuclei segmentation, which is carried out by a global thresholding and watersheding, foci segmentation is implemented with more accurate steps, namely:

1. calculate the threshold $T_{ot}$ of the image by Otsu's method. Otsu's method assumes that the image contains two classes of pixel intensities (foci and background) and searches for the threshold that minimizes the intra-class variance. The intra-class variance is defined as a weighted sum of variances of two classes [2]. For the image from Figure 2N $T_{ot} = 0.067$.
2. suppress all peaks of the image in three-dimensional representation whose height is less than some value *h* using H-maxima transform [2]. The height *h* of the peak is

defined as a vertical distance between the top of the peak and the closest valley (see Figure S2).

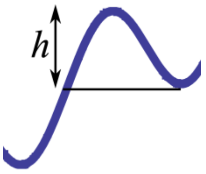


**Figure S2**. **The height of the peak** *h* is defined as a vertical distance between the top of the peak and the closest valley.

We set $h = T_{ot}$ (Figure 2O). The higher the value of the Otsu's threshold $T_{ot}$ is, the higher the peaks to be considered as foci. Thus, we get a reasonable estimate of the minimal height of a focus peak and avoid additional user-defined parameters for the algorithm.

3. find regional maxima of the resulting image (Figure 2P). Regional maxima are connected components of pixels with a constant value, and whose external boundary pixels all have lower values [2]. Thus, we get a binary mask with white pixels, where regional maxima are identified, and black pixels elsewhere (see Figure 2P).
4. apply the mask to the image obtained after the top-hat transform and threshold the result with the estimated threshold value $T_e$. This step is used to exclude those peaks, which height is larger than $T_{ot}$, but still not sufficiently large to consider them as foci. The '*threshold value*' $T_e$ is the fifth user-defined parameter. For the example image we used $T_e = 0.07$ (see Figure 2Q). We recommend an optimization procedure for the parameter $T_e$ in Supplementary Section S3.

Finally, after these segmentation steps we obtain the foci mask and marked the identified foci on the original image by red frames (see Figure 2R).

## S2. H-maxima transform

The aim of the H-maxima transform is to suppress all peaks of the three-dimensional intensity image (Figure 2N) whose height is less than *h* [2]. The height of the peak is defined as a vertical distance between the top of the peak and the closest valley (see Figure S2).

For demonstration, we consider a two-dimensional curve (black line in Figure S3). The abscissa of the plot mimics one of two dimensions in the image matrix that defines a pixel position. The ordinate mimics the pixel intensity value.

Now, we apply the H-maxima transform to suppress intensity peaks that have height less than 3. These peaks are designated by grey frames in Figure S3. The H-maxima transform implements the following steps:

1. create a marker curve by subtracting $h = 3$ from the original curve (blue curve in Figure S3).
2. dilate peaks of the marker curve using a 2-connected neighborhood (see Figure S4A) until the marker curve reaches the original curve. In Figure S3 the blue curve is dilated along blue dashed lines. The dilated peaks are displayed as red line in Figure S3.

Note, that the H-maxima transform not only suppressed peaks with the height less than 3, but also decreased remaining peaks.

Positions of regional maxima of the H-maxima transform are represented as magenta projections of regional maxima (Figure S3) and include pixels with positions $\{\{1,2,3\},\{6\},\{12\},\{15,16,17\}\}$.

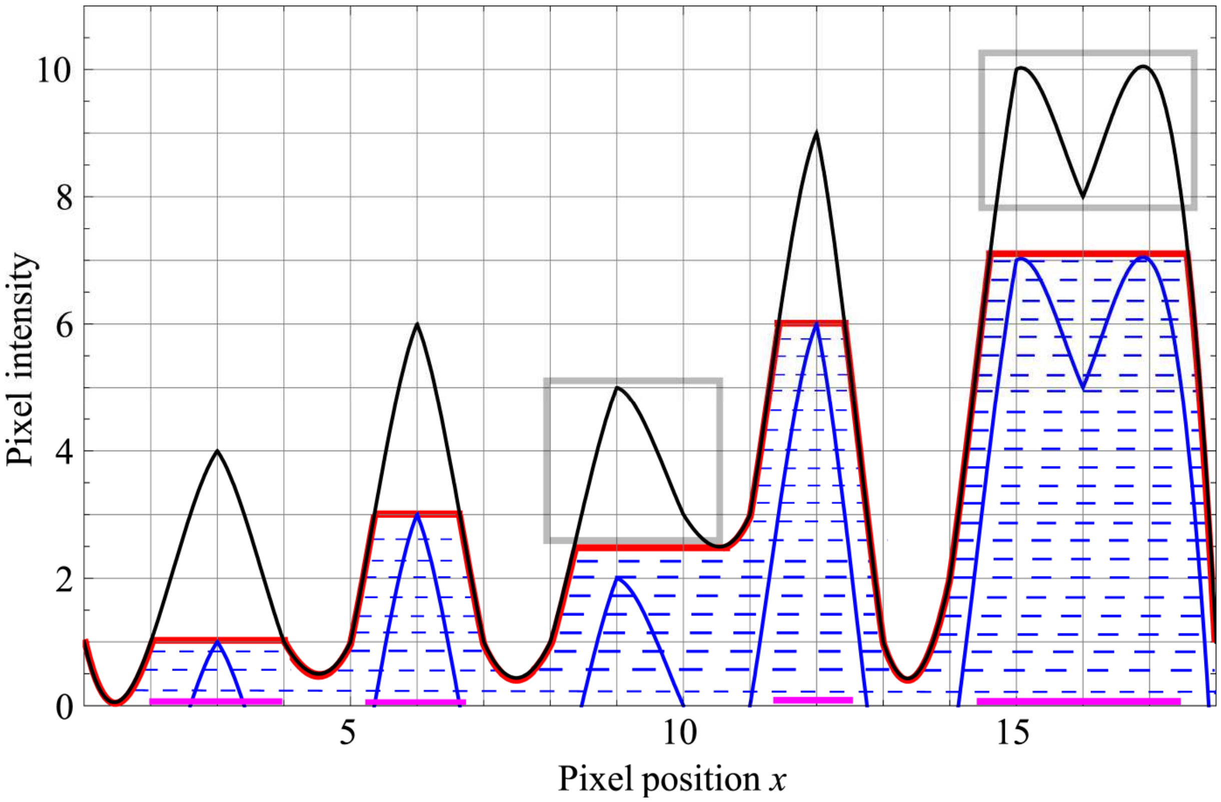


**Figure S3**. **Visualization of H-maxima transform applied to the black curve to suppress peaks with height less or equal to $h = 3$.**

The H-maxima transform of the image in three-dimensional representation (see Figure 2O) is performed in a similar way: the dilation of the marker image is performed using the 8-connected neighborhood parallel to the coordinate plane (see Figure S4B).

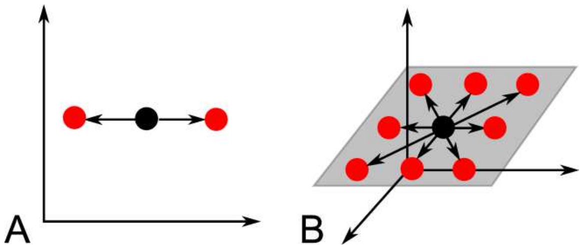


**Figure S4**. **Neighborhoods used for dilation.** **(A)** 2-connected neighborhood. **(B)** 8-connected neighborhood.

## S3. Optimization of parameter values $r_f$ and $T_e$: example with confocal microscope images

In this section, we propose an algorithm for the optimization of the user-defined parameter values $r_f$ and $T_e$. The first step is based on a previously published algorithm [4]. The principal goal of the parameter optimization is to maximize the number of identified induced foci and at the same time to minimize the number of identified background foci. To this end, a set of images is chosen that represent both maximal number of induced foci and background foci. Here, we chose images of cells, which were obtained 1 hour after irradiation with doses

of 2.5 and 10 Gy assuming that they have a high number of foci. In addition, we collected images of control cells, which were not exposed to irradiation, and assume that they show background foci only.

According to [4], we formulated the following optimization problem of finding parameters $(r_f, T_e)$ that maximize the difference of induced and background foci: Find $D_{\max}$ with respect to parameter values $(r_f, T_e)$:

$$D_{\max} = \max_{(r_f, T_e)} d = (N_{10Gy} - N_c) + (N_{2.5Gy} - N_c). \tag{1}$$

$N_{10Gy}$ and $N_{2.5Gy}$ correspond to the mean foci number per nucleus for cells 1 hour after 2.5 Gy and 10 Gy irradiation, respectively; $N_c$ corresponds to the mean foci number per nucleus for control cells.

We solved (1) numerically by calculating $N_{10Gy}$, $N_{2.5Gy}$, $N_c$ and $d = (N_{10Gy} - N_c) + (N_{2.5Gy} - N_c)$ for all possible combinations of parameters $(r_f, T_e)$, where $r_f \in \{2,3,4,5\}$ and $T_e \in \{0.05, 0.06, 0.07, 0.08, 0.09\}$ (Figure S5A). The set of parameter values for $r_f$ and $T_e$ is defined by the operator after performing a series of test foci quantifications in FoCo. The range of parameters should be chosen such that foci can be identified according to visual inspection.

Here, the tuple $(r_f, T_e) = (2, 0.06)$ is optimal in the sense of Eq. (1) (Figure S5A). However, this step alone is often not sufficient for the optimization of foci count for the control sample. Note that Eq. (1) takes into account absolute values of $N_{10Gy}$, $N_{2.5Gy}$, and $N_c$. Naturally, absolute induced foci number ($N_{10Gy}$, $N_{2.5Gy}$) is usually much higher than background foci number ($N_c$) (Figure S6). Consequently, maximizing induced foci number has a stronger influence on optimal parameter selection than minimizing background foci number. Therefore, we extend the algorithm proposed in [4] by applying a second step to improve minimization of the background foci count.

Thus, in the second step, for each value of $r_f \in \{2,3,4,5\}$ we find a value of $T_e$ that maximizes $d$ (Figure S5A): $(r_f, T_e) = (2, 0.06)$, $(r_f, T_e) = (3, 0.07)$, $(r_f, T_e) = (4, 0.07)$, $(r_f, T_e) = (5, 0.07)$. Then, for each obtained pair $(r_f, T_e)$, we quantify $N_c$ (Figure S5B). Finally, the pair of parameters that minimizes $N_c$ is defined to be optimal. Here the optimal parameter set is $(r_f, T_e) = (3, 0.07)$.

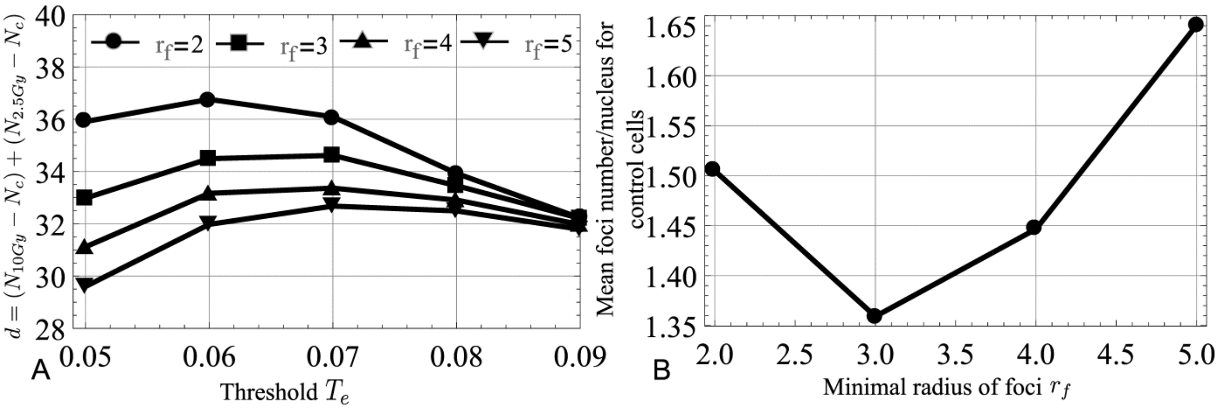


**Figure S5. Steps of the algorithm for the optimization of parameter values $T_e$ and $r_f$. (A)** Values of $d = (N_{10Gy} - N_c) + (N_{2.5Gy} - N_c)$ for sampled pairs $(r_f, T_e)$. **(B)** Quantification of $N_c$ for parameter pairs $(r_f, T_e) = (2, 0.06)$, $(r_f, T_e) = (3, 0.07)$, $(r_f, T_e) = (4, 0.07)$, $(r_f, T_e) = (5, 0.07)$, which maximize $d$ for each value of $r_f$.

Some special cases still have to be covered:

1) In case there is no unique parameter pair with minimal $N_c$ among those having $D_{\max}$ for all $r_f$, then the parameter pair with maximal $D_{\max}$ is defined to be optimal.
2) In case there is no unique parameter pair for $D_{\max}$, the one with minimal $N_c$ is defined to be optimal.
3) In case there are several parameter pairs with the same maximal $D_{\max}$ and minimal $N_c$, than anyone can be defined to be optimal.

For demonstration of foci quantification with optimal parameters we depicted in Figure S7 representative nuclei with detected foci in FoCo at time points 1, 3, 6, 24, 72, 168 hours after 2.5 Gy and 10 Gy irradiation. Additionally, in Figure S8 we demonstrated results of manual and automatic foci detection applied to the cropped part of the image, which was obtained 3 hours after 10 Gy irradiation and contains several nuclei (Figure S8A). Figure S8C presents foci detection in FoCo.

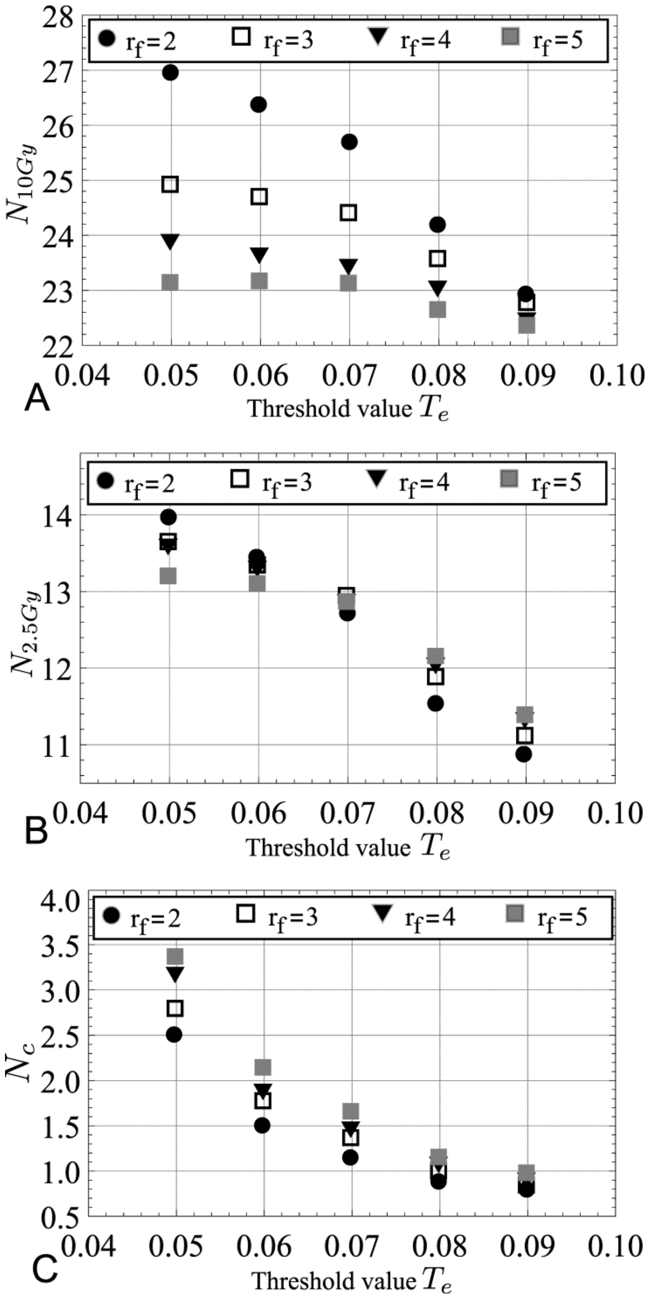


**Figure S6. Automatic quantification of mean foci numbers per nucleus in FoCo for sampled parameter values** $r_f \in \{2,3,4,5\}$ and $T_e \in \{0.05, 0.06, 0.07, 0.08, 0.09\}$ used for calculating $d = (\mathrm{N}_{10Gy} - \mathrm{N}_c) + (\mathrm{N}_{2.5Gy} - \mathrm{N}_c)$ presented in Figure S5A. **(A)** $N_{10Gy}$ corresponds to the mean foci number per nucleus for cells after 1 hour after 10 Gy irradiation. **(B)** $N_{2.5Gy}$ corresponds to the mean foci number per nucleus for cells after 1 hour after 2.5 Gy irradiation. **(C)** $N_c$ corresponds to the mean foci number per nucleus for control cells.

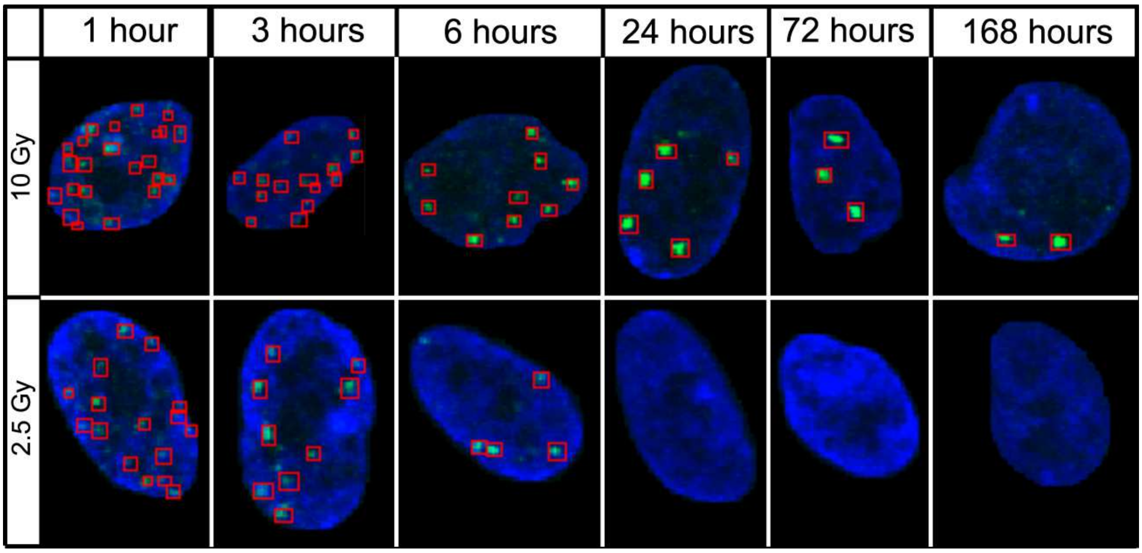


**Figure S7. Representative nuclei (blue) with γH2AX foci (green) from the image set obtained on confocal laser-scanning fluorescent microscope** at time points 1, 3, 6, 24, 72, 168 hours after 2.5 Gy and 10 Gy irradiation. Foci detected by FoCo are designated by red frames.

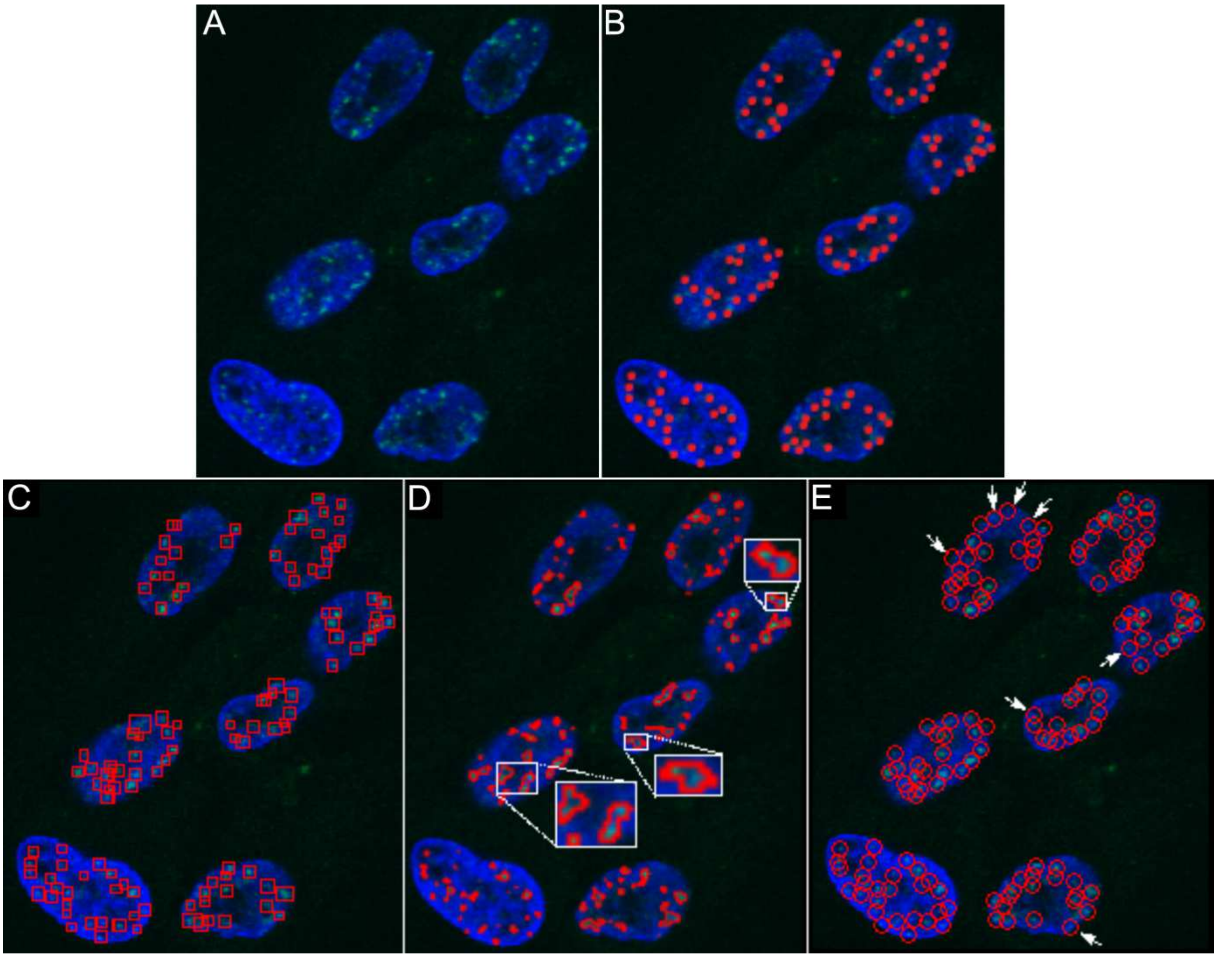


**Figure S8. Demonstration of manual and automatic foci quantifications. (A)** The image obtained 3 hours after 10 Gy irradiation on confocal laser-scanning fluorescent microscope. **(B)** Results of manual foci quantifications by one of operators. Detected foci are marked by red dots. **(C)** Quantifications in FoCo. Detected foci are designated by red frames. **(D)** Quantifications in CellProfiler. Detected foci are designated by red outlines. Enlarged regions

visualize the disability of the CellProfiler algorithm to separate foci, which are located close to each other. **(E)** Quantifications in ImageJ. Detected foci are marked by red circles. White arrows point at detected foci, which have very low signal and from the visual perspective belong to background noise.

## S4. Optimization of parameter values $r_f$ and $T_e$: example with wide-field microscope images

In order to detect cell nuclei on images obtained on wide-field fluorescent microscope, we utilized the same parameters, which we used for nuclei detection on images obtained on confocal laser-scanning microscope (see section S3).

For the optimization of parameter values $T_e$ and $r_f$, we followed the algorithm presented in the section S3. First, we selected images of cells obtained 1 hour after 10 Gy radiation and images of control cells. Then, we define *d* with

$$d = (\mathrm{N}_{10Gy} - \mathrm{N}_c), \quad (2)$$

where $N_{10Gy}$ corresponds to the mean foci number per nucleus for cells after 1 hour after 10 Gy irradiation; $N_c$ corresponds to the mean foci number per nucleus for control cells.

Then, for each $r_f \in \{4,5,6,7\}$ we find $T_e \in \{0.011, 0.014, 0.017, 0.023, 0.026, 0.029\}$ that maximizes $d$ (Figure S9A): $(r_f, T_e) = (4, 0.014)$, $(r_f, T_e) = (5, 0.017)$, $(r_f, T_e) = (6, 0.026)$, $(r_f, T_e) = (7, 0.026)$. For each of these tuples we quantified $N_c$ (Figure S9B). As the optimal parameter pair we identified the tuple $(r_f, T_e) = (6, 0.026)$, which minimizes $N_c$.

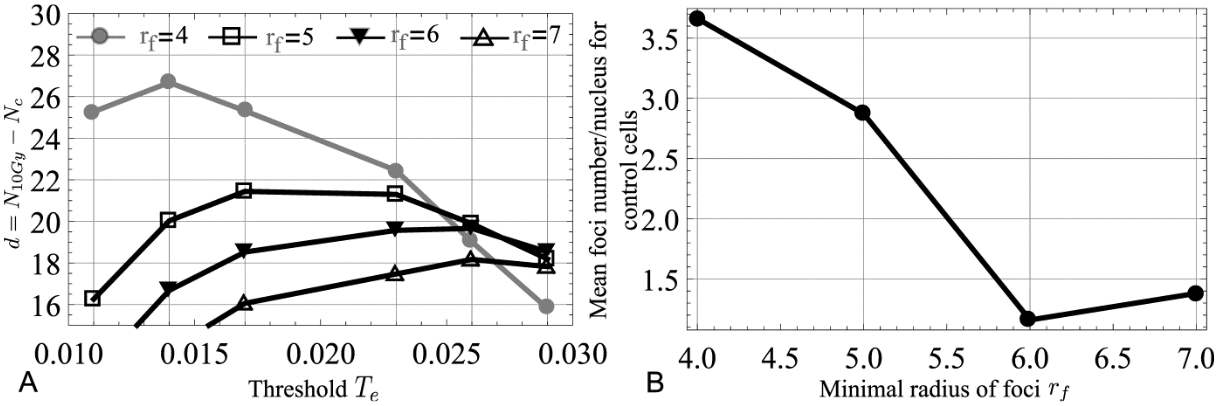


**Figure S9. Steps of the algorithm for the optimization of parameter values $T_e$ and $r_f$. (A)** Calculation of $d = \mathrm{N}_{10Gy} - \mathrm{N}_c$ for sampled parameter values $(r_f, T_e)$. **(B)** Quantification of $N_c$ for parameter pairs $(r_f, T_e) = (4, 0.014)$, $(r_f, T_e) = (5, 0.017)$, $(r_f, T_e) = (6, 0.026)$, $(r_f, T_e) = (7, 0.026)$, which maximize $d$ for each value of $r_f \in \{4,5,6,7\}$.

In Figure S10 we demonstrate representative nuclei with detected foci at time points 1, 3, 6, 24, 72 hours after 10 Gy irradiation. For foci detection we used optimal parameters obtained in this section. One may notice the difference in the quality of images taken on confocal laser-scanning and wide-field fluorescent microscopes (Figure S7, S8 and Figure S10):

images from the wide-field fluorescent microscope have elevated background for the green signal. Consequently, foci look blurred and are not easily distinquishable by eye.

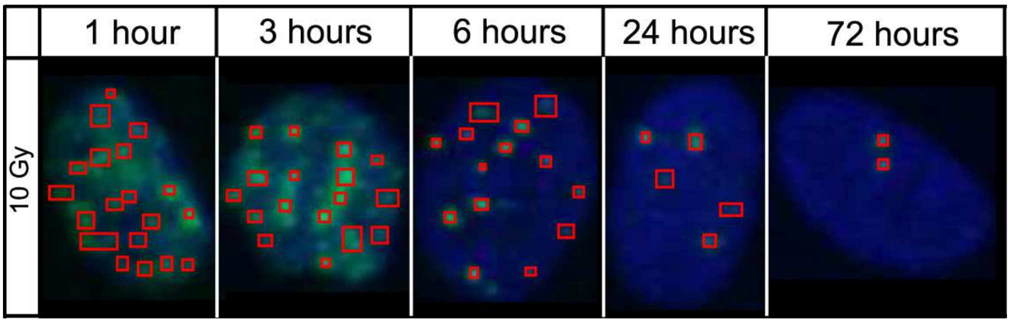


**Figure S10. Representative nuclei (blue) with γH2AX foci (green) from the image set obtained on wide-field fluorescent microscope** at time points 1, 3, 6, 24, 72 hours after 10 Gy irradiation. Foci detected by FoCo are designated by red frames.

## S5. Optimization of parameter values for CellProfiler: example with confocal microscope images

The CellProfiler pipeline, which was applied for foci quantification on images obtained with a confocal laser-scanning microscope, contains more than 10 parameters needed for nuclei and foci detection. Therefore, the majority of parameter values were optimized manually by performing a series of test quantifications.

Some parameter values, which to our opinion have a major impact on foci counting, were optimized in a similar manner as parameter $r_f$ and $T_e$ for FoCo. These are the parameters for foci detection '*Typical diameter of foci*' and '*Thresholding method*'. After performing test quantifications, for '*Typical diameter of foci*' we considered two parameter combinations for the *minimal diameter* and *maximal diameter*, i.e. {1,7} and {1,10}, respectively. Again, after performing test quantifications, we considered '*Manual*' as '*Thresholding method*' with threshold values 0.07, 0.08, 0.09, 0.1, 0.11, 0.12, 0.13, and 0.14.

As in the section S3, for each '*Typical diameter of foci*'-combination we calculated $d = (\mathrm{N}_{10Gy} - \mathrm{N}_c) + (\mathrm{N}_{2.5Gy} - \mathrm{N}_c)$ for all threshold values, selected the two '*Typical diameter of foci*'-threshold pairs with maximal *d*, and defined the one with minimal $N_c$ as optimal. The optimal '*Typical diameter of foci*'-threshold resulted to be {{1,7},0.11}. $N_{10Gy}$, $N_{2.5Gy}$ and $N_c$ are defined as in section S3. Note that for quantifications we used the same images that we used for calculating $N_{10Gy}$, $N_{2.5Gy}$ and $N_c$ in FoCo and ImageJ.

In Figure S8D we demonstrated the application of CellProfiler with optimized parameters for quantifying foci on the representative image of cells 3 hours after 10 Gy irradiation. In enlarged regions we visualized the disability of the algorithm to separate foci, which are located close to each other.

## S6. Optimization of parameter values for ImageJ: example with confocal microscope images

Firstly, we present macros, which we used for performing foci quantifications in ImageJ:

```
/*Load the image for analysis*/
open("E:\\Original.tif");
```

```
run("Split Channels");
/*Detect nuclei*/
selectWindow("Original.tif (blue)");
setAutoThreshold("Huang");
//run("Threshold...");
//setThreshold(0, 22);
setOption("BlackBackground", false);
run("Make Binary", "thresholded remaining black");
run("Convert to Mask");
run("Fill Holes");
run("Watershed");
run("Analyze Particles...", "size=200-infinity pixel show=[Overlay Outlines] display exclude add");
roiManager("Show All with labels");
roiManager("Show All");
/*Detect foci*/
selectWindow("Original.tif (green)");
run("Find Maxima...", "noise=14 output=[Single Points]");
roiManager("Show None");
roiManager("Show All");
roiManager("Measure");
saveAs("Results", "E:\\Results.xls");
```

ImageJ utilizes only three parameters: two parameters for nuclei detection, namely, 'Thresholding method' for nuclei images and 'Size' of nuclei, and one parameter for foci detection 'Noise tolerance'.

Parameters for nuclei detection were adjusted manually by performing several test quantifications. For foci detection we sampled the value of the parameter 'Noise tolerance' in the range between 14 and 25. Then for each sampled value we quantified foci on images of cells 1 hour after 2.5 Gy and 10 Gy irradiation, respectively, and for images of control cells. Correspondingly, we obtained values $N_{10Gy}$, $N_{2.5Gy}$ and $N_c$ (see Figure S11A). Further, we calculated the difference $d = (\mathrm{N}_{10Gy} - \mathrm{N}_c) + (\mathrm{N}_{2.5Gy} - \mathrm{N}_c)$ (see Figure S11B). Note that for quantifications we used the same images that we used for calculating $N_{10Gy}$, $N_{2.5Gy}$ and $N_c$ in FoCo and CellProfiler.

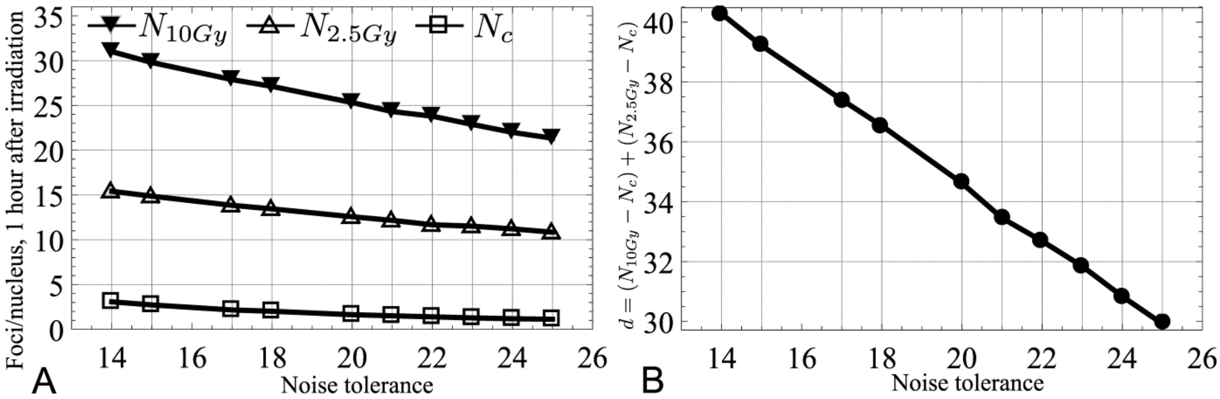


**Figure S11. Optimisation of the parameter value 'Noise tolerance'** needed for the ImageJ algorithm and sampled in the range between 14 and 25. **(A)** Quantification of the mean foci number per nucleus for images of cells 1 hour after 2.5 Gy and 10 Gy and control cells, which correspond to $N_{10Gy}$, $N_{2.5Gy}$ and $N_c$, respectively. **(B)** The calculated difference $d = (\mathrm{N}_{10Gy} - \mathrm{N}_c) + (\mathrm{N}_{2.5Gy} - \mathrm{N}_c)$.

In contrast to quantifications in CellProfiler and FoCo, *d* is monotonically decreasing with 'Noise tolerance' in the considered interval (Figure S7B). Thus, the minimal value for 'Noise tolerance', i.e. 14, defines the optimum, as it provides the maximum value of *d.*

For demonstration of foci quantification in ImageJ with the optimal 'Noise tolerance' we utilized a representative cropped image of cells 3 hour after 10 Gy irradiation (see Figure S8E). With white arrows we pointed at detected foci, which have very low signal and from visual perspective belong to the background noise.

## S7. Outlier detection

For several reasons, e.g., problems with foci staining, low resolution of the microscope, algorithm constraints, etc., some nuclei can have exceptionally high or low number of foci, compared to other nuclei as the same time point. These are considered as outliers. In order to detect and eliminate outliers we apply the following procedure:

- quantify the first $Q_1$ and third $Q_3$ quartiles and interquartile range $IQR = Q_3 - Q_1$ of the number of foci per nucleus per time point.
- quantify upper *(UL)* and lower *(LL)* limits:

$$LL = Q_1 - 1.5IQR,$$
$$UL = Q_3 + 1.5IQR.$$

- consider the nucleus as outlier if its number of detected foci $N \le LL$ or $N \ge UL$.

## S8. Manual foci quantifications

Three operators (one with and two without professional experience in biology) performed independent manual foci counting. Operators were not informed about the results of automatic counting. Moreover, the operators were not aware of time points and radiation regimes corresponding to the images. Manual counting results were available to the operator performing automatic counting after finishing automatic count and were not used for optimization of algorithmic parameters.

## S9. Comparison between automatic and manual foci quantifications

Despite existing numerous computational approaches, manual counting still remains the gold standard for foci counting [4, 5] and is used for validation of automatic foci quantifications [4-9]. Therefore, we wondered how foci quantifications in FoCo, CellProfiler and ImageJ would correspond to manual quantifications.

Since manual foci quantification is laborious and time-consuming, we did not perform manual count for the whole image set obtained with the confocal laser-scanning microscope. Instead, we formed a subset of 16 representative images, which we called a test image set. The test image set contained one image of control cells for each time point 1, 24, 72, 168 hours and one or two images of cells post 2.5 Gy and 10 Gy for each time point 1, 3, 6, 24, 72, 168 hours. Then, the test image set was subjected to manual quantification by three operators (Manual 1, Manual 2 and Manual 3) and automatic quantification in FoCo, CellProfiler and ImageJ. Note that for the automatic quantification of the test image set we used the same parameters as for processing of the whole image set.

Results of automatic quantification of the test image set in FoCo, ImageJ and CellProfiler are represented in Figure S12A,B and C, respectively. Manual quantifications of the test image set Manual 1, Manual 2 and Manual 3 are presented in Figure S12D,E and F, respectively.

Interestingly, all manual quantifications show that mean foci number per nucleus is decreasing in time despite the fact that the operators did not know about the respective time points. However, they differ significantly in absolute values at time points 1, 3 and 6 hours after irradiation (see Figure S12D, E). This demonstrates a subjectivity of manual quantification, which can be explained by the ambiguity in distinguishing between actual foci and background shortly after irradiation.

Quantification results obtained by automatic methods also differ from each other. According to quantification in FoCo, the mean foci number per nucleus is decreasing in time after DNA damage for both 2.5 Gy and 10 Gy samples. However, quantifications in

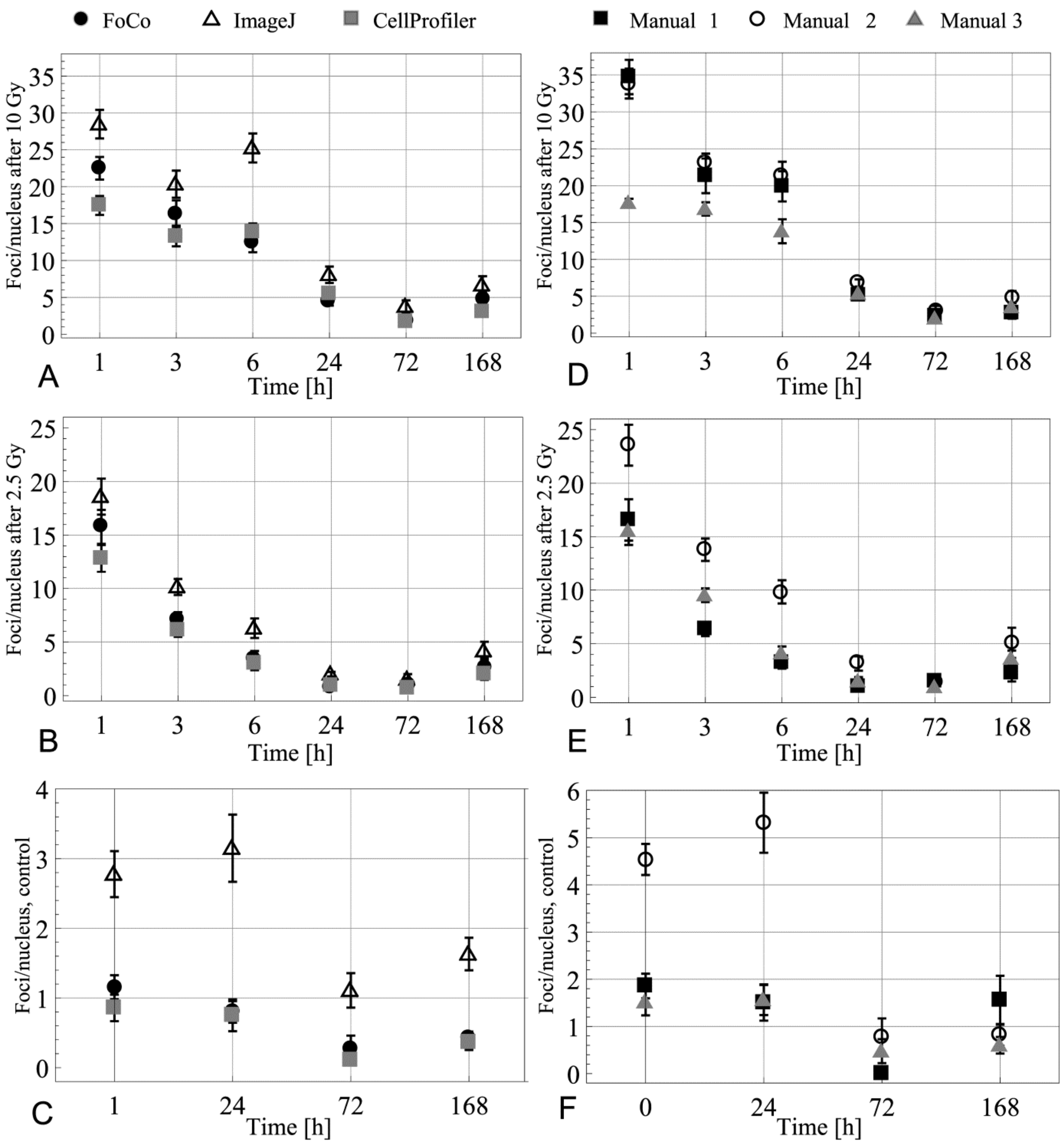


**Figure S12**. **Results of $\gamma H2AX$ foci quantification for the test image set** using automatic **(A-C)** and manual **(D-F)** approaches. **(A, D)** Mean foci number per nucleus after 10 Gy; **(B, E)** Mean foci number per nucleus after 2.5 Gy; **(C, F)** Mean foci number per nucleus for control cells. Error bars represent SEM (n≥10).

CellProfiler and ImageJ show that the mean foci number per nucleus is monotonically decreasing with time only for 2.5 Gy sample and has a peak at the time point 6 hours for 10 Gy sample. As for manual count, there is also a significant difference in absolute values of mean foci numbers per nucleus for time points 1, 3 and 6 hours after irradiation. For control sample the mean number of foci stays approximately constant for FoCo and CellProfiler. However, for ImageJ the mean number of foci per nucleus varies between 1 and 3 foci per nucleus and is about twice higher than for other methods.

**Weighted orthogonal regression analysis**

To compare manual and automatic foci quantifications we fitted a linear relationship between manual and automatic foci counts for each radiation regime and each combination of operator and automatic method, respectively, by a weighted orthogonal regression (Figure S13). The null hypothesis assumed a 1:1 linear relationship between manual and automatic quantifications, i.e., $H_0 : a + bx$ with $(a,b) = (0,1)$, designated by a dot-dashed line in Figure S13. In the left part of Figure S13A-C we depicted results of the regression analysis between automatic quantifications in FoCo (black line), ImageJ (dark grey line), CellProfiler (light grey line) and Manual 1 for 10 Gy, 2.5 Gy and control, respectively. The regression analysis between automatic quantifications and Manual 2 and Manual 3 is performed in the analogous way.

Further, we calculated 25, 50, 75, 90 and 95% confidence regions using a Monte-Carlo approach, sampling 10000 times from a Gaussian distribution within the standard error of the mean of respective manual and automatic counts. We tested $H_0$ by assigning the estimated parameter pairs $(a,b)$ to the respective confidence region (right part of Figure S13D-K). The estimated parameter pairs and the hypothesized pair $(a,b) = (0,1)$ are designated by black and grey dots, respectively.

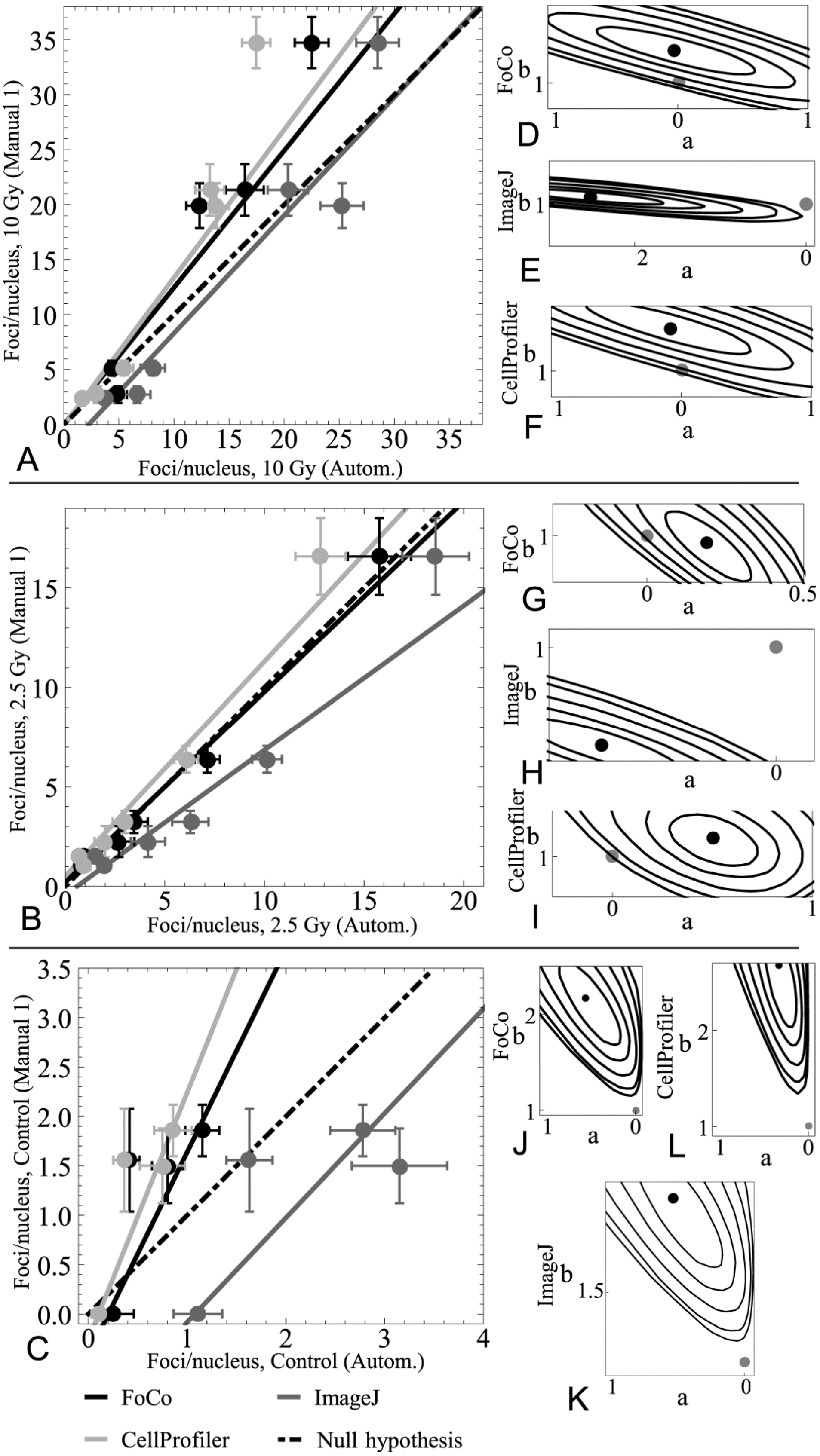


**Figure S13**. **Results of weighted orthogonal regression analysis** between manual quantifications Manual 1 and automatic quantifications in FoCo, Cellprofiler and ImageJ performed for 10 Gy sample **(A)**, 2.5 Gy sample **(B)** and control sample **(C)**. The null hypothesis (dot-dashed line) assumed a 1:1 linear relationship between automatic and

manual quantification results, i.e., $H_0 : a + bx$ with $(a,b) = (0,1)$. Based on weighted orthogonal regression, we estimated actual parameter pairs $(a,b)$ and depicted obtained relations in the left part of the figure (black line for FoCo, dark grey for ImageJ and light grey for CellProfiler). Dots designate mean foci number per nucleus. Further, using Monte-Carlo analysis we computed 25, 50, 75, 90 and 95% confidence regions of actual parameter pairs. Then for each sample and for each automatic method we defined a confidence region, within which the hypothesized pair $(a,b) = (0,1)$ designated by grey dot is located (see the right part of the figure). Error bars represent SEM (n≥10).

Table S1 demonstrates if the hypothesized pair $(a,b) = (0,1)$ is located within the 95% confidence region of the actual estimated parameter pair for performed automatic and manual foci quantifications.

Interestingly, quantification results in FoCo demonstrated the hypothesized relationship to Manual 3 within the 95% confidence region for all datasets 10 Gy, 2.5 Gy and control (Table S1). This indicates a high correlation between automatic quantification in FoCo and Manual 3. Manual 1 is correlated equally high to both FoCo and CellProfiler. Manual 2 correlated badly to all automatic quantification results.

**Table S1. Results of weighted orthogonal regression analysis.** The analysis was performed to reveal a correlation between automatic (FoCo, CellProfiler, ImageJ) and manual (Manual 1, Manual 2, Manual 3) quantification results. We checked if the hypothesized pair $(a,b) = (0,1)$, which represents a 1:1 linear relationship between manual and automatic quantifications, is located within 95% confidence region (+) or not (-) of the actual estimated parameter pair.

| | FoCo | CellProfiler | ImageJ |
|---|---|---|---|
| | Manual 1 | | |
| 10 Gy | + | + | - |
| 2.5 Gy | + | + | - |
| Control | - | - | - |
| | Manual 2 | | |
| 10 Gy | - | - | + |
| 2.5 Gy | - | - | - |
| Control | - | - | - |
| | Manual 3 | | |
| 10 Gy | + | + | - |
| 2.5 Gy | + | - | + |
| Control | + | + | - |

### Spearman rank correlation

Further, we calculated Spearman rank correlation coefficient, which estimates how well the relationship between manual and automatic foci quantifications can be described using a monotonic function. Namely, Spearman rank correlation shows whether, as mean foci number per nucleus according to manual counting decreases in time, the mean foci number per nucleus according to automatic counting tends to decrease in time as well. A Spearman correlation of 1 occurs when manual and automatic quantifications are perfectly monotonically related. The lower the Spearman correlation coefficient, the more non-monotonic relation manual and automatic quantifications demonstrate.

The results of Spearman rank correlation between manual and automatic quantifications of the test image set are demonstrated in Table S2. Thus, Spearman rank correlation coefficient for FoCo and manual quantifications is greater than 0.8 for non-irradiated and irradiated samples, whereas the lowest Spearman rank correlation coefficients for CellProfiler and ImageJ have values 0.77 and 0.4, respectively.

**Table S2. Results of Spearman rank correlation.** Spearman rank correlation was performed for revealing a monotonic relation between manual (Manual 1, Manual 2 and Manual 3) and automatic (FoCo, CellProfiler and ImageJ) foci quantification results obtained for the test image set.

| | FoCo | CellProfiler | ImageJ |
|---|---|---|---|
| | Manual 1 | | |
| 10 Gy | 0.94 | 0.94 | 0.94 |
| 2.5 Gy | 1 | 0.77 | 0.94 |
| Control | 0.8 | 0.8 | 0.4 |
| | Manual 2 | | |
| 10 Gy | 0.94 | 0.94 | 0.94 |
| 2.5 Gy | 0.94 | 1 | 1 |
| Control | 0.8 | 0.8 | 1 |
| | Manual 3 | | |
| 10 Gy | 0.94 | 0.94 | 0.94 |
| 2.5 Gy | 0.94 | 1 | 1 |
| Control | 0.8 | 0.8 | 1 |

To summarize, results of two statistical approaches show a high correlation between automatic quantifications in FoCo and Manual 1 and Manual 3. This is the best result for considered automatic methods.

## S10. Simulation and analysis of simulated foci images with pre-defined number of foci

Since all considered automatic approaches detected approximately the same number of cell nuclei for all analyzed time points and eventually average over detected nuclei (Table S3), we omitted simulation of nuclei images and simulated only foci images. Thus, we assume that every simulated grayscale foci image does not need a nuclei mask and belongs to one nucleus.

**Table S3.** The number of nuclei detected by FoCo, CellProfiler and ImageJ in the image set obtained on confocal laser scanning microscope.

| | FoCo | CellProfiler | ImageJ |
|---|---|---|---|
| Time [h] | | 10 Gy | |
| 1 | 103 | 107 | 105 |
| 3 | 127 | 133 | 130 |
| 6 | 98 | 105 | 104 |
| 24 | 107 | 109 | 108 |
| 72 | 102 | 105 | 103 |
| 168 | 94 | 108 | 107 |
| | | 2.5 Gy | |
| 1 | 128 | 139 | 139 |
| 3 | 132 | 138 | 139 |

| | | | |
|---|---|---|---|
| 6 | 102 | 112 | 105 |
| 24 | 99 | 106 | 103 |
| 72 | 112 | 127 | 125 |
| 168 | 103 | 113 | 108 |
| | | Control | |
| 1 | 103 | 110 | 109 |
| 24 | 113 | 110 | 109 |
| 72 | 110 | 111 | 110 |
| 168 | 125 | 128 | 125 |

For simulating artificial foci images we created a range of focus templates (Figure S14):

- Using a normal distribution with different standard deviations $\sigma$ we created single foci as peaks with height 1. We randomly varied $\sigma$ in the certain range for each focus template to simulate foci o different size: $\sigma = 0.3 - 0.7$ (Figure S14A), $\sigma = 0.8 - 1.2$ (Figure S14B), $\sigma = 1.3 - 1.7$ (Figure S14C), $\sigma = 2.8 - 3.2$ (Figure S14D).
- We scaled the focus template from Figure S14B with the random factor from the range of 0.6 to 0.8 and combined it with the focus template from Figure S14D. This way we mimicked two distinct foci of different sizes touching each other (Figure S14E). We also combined two foci templates presented in Figure S14C (Figure S14F). This way we mimicked two distinct foci of approximately the same size touching each other.
- We scaled the focus template from Figure S14B with a random factor from the range of 0.2 to 0.5 and put it close to the focus template from Figure S14D. The resulting focus template is presented in Figure S14G. In addition, we overlapped two foci templates presented in Figure S14C (Figure S14H). Both foci templates from Figure S14G,H mimic one focus with the presence of some noise.

Thus, we obtained eight different foci templates presented in Figure S14 A-H, which mimic either one (A-D, G,H) focus or two (E,F) foci.

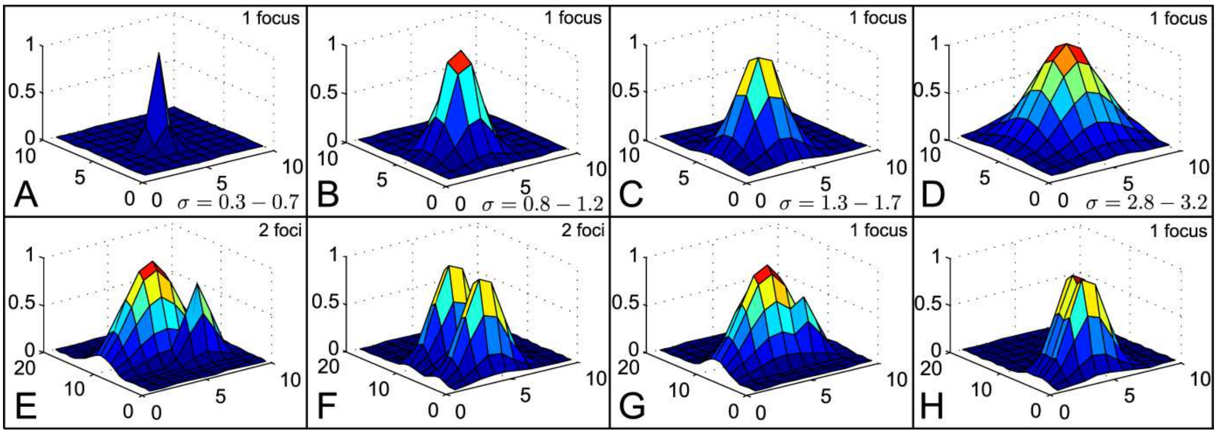


**Figure S14**. **Focus templates for simulating foci images.** **(A-D)** Single foci generated by the normal distribution with different standard deviations $\sigma$. **(E)** Combination of the scaled focus template from panel B and D. The scaling factor varies randomly in the range of 0.6 to 0.8. **(F)** Combination of two foci templates from panel C. Focus templates (E-F) mimic two distinct foci touching each other. **(G)** Combination of the scaled focus template from panel B and D. The scaling factor varies randomly in the range from 0.2 to 0.5. **(H)** Combination of

two focus templates from panel C. Focus templates (G-H) mimic one focus with the presence of some noise.

The simulation of the foci image consists of two steps: placing actual foci on the empty image and applying a background noise. For the first step, we randomly sampled a certain number of focus templates from Figure S14A-H (50 for irradiated images and 5 for control images), scaled each of them with the random factor from the range of 0.8 to 1 and randomly placed them on the empty image of the size 100x100.

Then, we added noise. To this end, we randomly sampled the same number of focus templates from Figure S14A-H (50 for irradiated images and 5 for control images), scaled each of them with the random factor from the range of 0.1 to 0.5 and randomly placed them on the image with actual foci. Then we create a matrix of the size 100x100 and randomly sample its values in the range of 0.1 to 0.2. Next we applied the maximum function pairwise to elements of the obtained random matrix and foci image with the background signal. Finally, we created a matrix of the size 100x100, randomly sample its values in the range of 0 to 0.1 and pairwise added its values to the image obtained in the previous step. The resulting image is a simulated foci image with the pre-defined number of foci (Figure 4C,D).

We used Matlab for simulating foci images (M-files CreateAnalyzeArtificialImages.m and peak.m in Additional file 6). Using coordinates of actual foci the program puts blue frames over placed foci (Figure S15A). This helps the user to distinguish between actual foci and background and perform benchmarking of automatic foci quantifications (Figure S15B-D).

Since we omitted simulation of nuclei images, we simplified algorithms of FoCo, ImageJ and CellProfiler by excluding nuclei detection part. The FoCo algorithm for analyzing simulated foci images was included in the M-file CreateAnalyzeArtificialImages.m with parameter values $(r_f, T_e) = (2, 0.18)$. In CellProfiler we utilized a pipeline with parameters {1,15} for the *minimal diameter* and *maximal diameter* and '*Manual*' as '*Thresholding method*' with the threshold value 0.4 (see Additional file 7). For analyzing images with ImageJ we used the parameter value 'Noise tolerance' equal to 25 and the following macros:

```
open("E:\\Image.tif");

run("Find Maxima...", "noise=25 output=Count");

saveAs("Results", "E:\\Results.xls");
```

For all considered algorithms we used parameter optimization procedures presented in sections S3-S6.

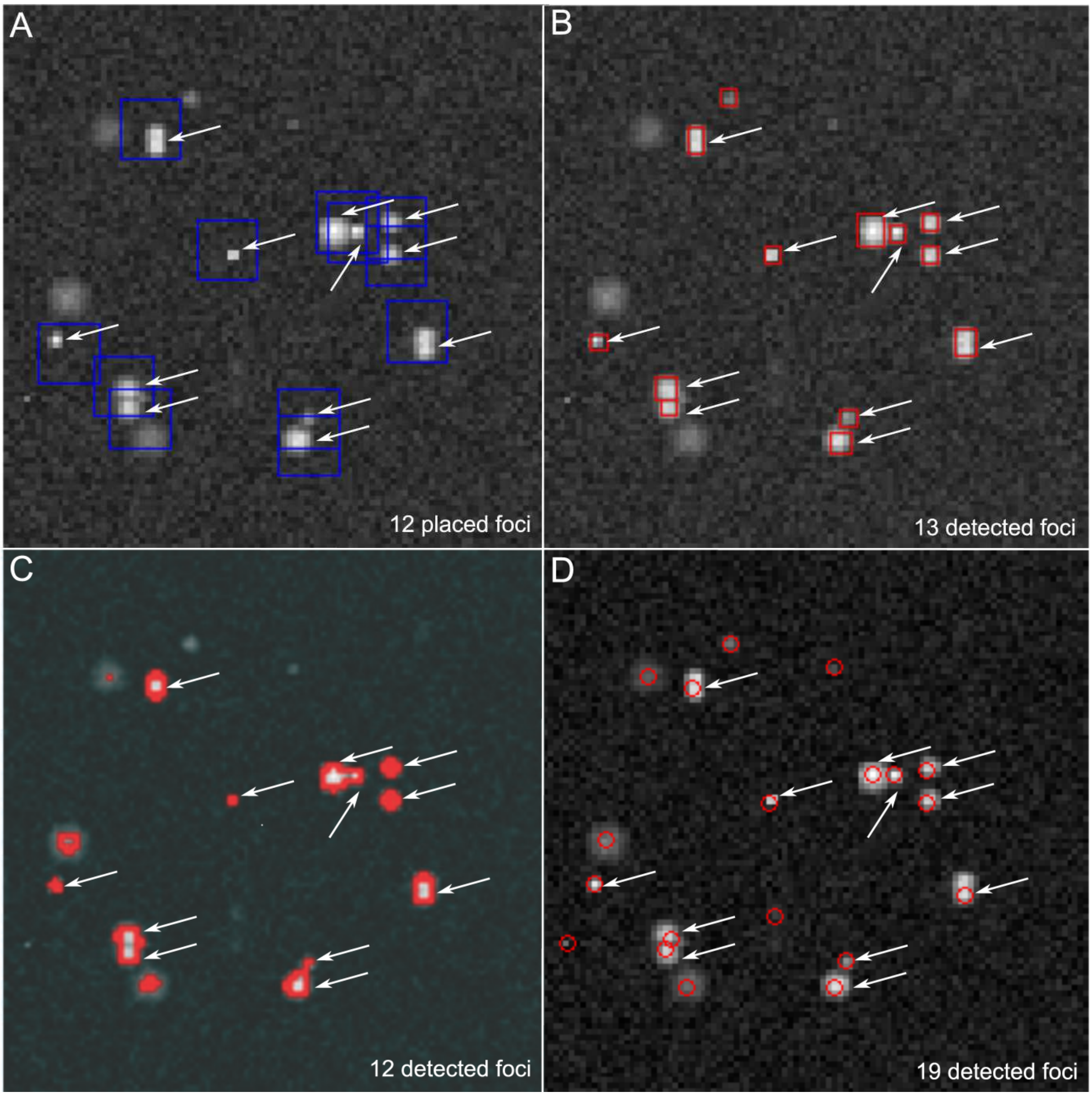


**Figure S15**. **Benchmarking automatic foci quantification by simulating foci images with the pre-defined foci number. (A)** Simulated foci image with 12 placed foci labeled with blue frames. **(B)** Simulated foci image with 13 detected foci in FoCo labeled with red frames. **(C)** Simulated foci image with 12 detected foci in CellProfiler with labeled foci boundaries. **(D)** Simulated foci image with 19 detected foci in ImageJ labeled with red circles. White arrows point at actual placed foci.

## References:


1. Jucha A, Wegierek-Ciuk A, Koza Z, Lisowska H, Wojcik A, Wojewodzka M, Lankoff A: **FociCounter: a freely available PC programme for quantitative and qualitative analysis of gamma-H2AX foci.** *Mutat Res* 2010, **696:**16-20.
2. Gonzalez RC, Woods RE, Eddins SLU: *Digital Image Processing Using MATLAB.* Pearson Prentice Hall; 2004.
3. Sedelnikova OA, Pilch DR, Redon C, Bonner WM: **Histone H2AX in DNA damage and repair.** *Cancer Biol Ther* 2003, **2:**233-235.

4. Ivashkevich AN, Martin OA, Smith AJ, Redon CE, Bonner WM, Martin RF, Lobachevsky PN: **gammaH2AX foci as a measure of DNA damage: a computational approach to automatic analysis.** *Mutat Res* 2011, **711:**49-60.
5. Anderson D, Andrais B, Mirzayans R, Siegbahn EA, Fallone BG, Warkentin B: **Comparison of two methods for measuring gamma-H2AX nuclear fluorescence as a marker of DNA damage in cultured human cells: applications for microbeam radiation therapy.** *Journal of Instrumentation* 2013, **8**.
6. Bocker W, Iliakis G: **Computational Methods for analysis of foci: validation for radiation-induced gamma-H2AX foci in human cells.** *Radiat Res* 2006, **165:**113-124.
7. Cai Z, Vallis KA, Reilly RM: **Computational analysis of the number, area and density of gamma-H2AX foci in breast cancer cells exposed to (111)In-DTPA-hEGF or gamma-rays using Image-J software.** *Int J Radiat Biol* 2009, **85:**262-271.
8. Dzyubachyk O, Essers J, van Cappellen WA, Baldeyron C, Inagaki A, Niessen WJ, Meijering E: **Automated analysis of time-lapse fluorescence microscopy images: from live cell images to intracellular foci.** *Bioinformatics* 2010, **26:**2424-2430.
9. Gonzalez JE, Lee M, Barquinero JF, Valente M, Roch-Lefevre S, Garcia O: **Quantitative image analysis of gamma-H2AX foci induced by ionizing radiation applying open source programs.** *Anal Quant Cytol Histol* 2012, **34:**66-71.
