# Supplementary figures and images for: FoCo: a simple and robust quantification algorithm of nuclear foci
Source: BMC Bioinformatics. 2015 Nov 21;16:392. doi: 10.1186/s12859-015-0816-5 (PMC4654864; doi:10.1186/s12859-015-0816-5)

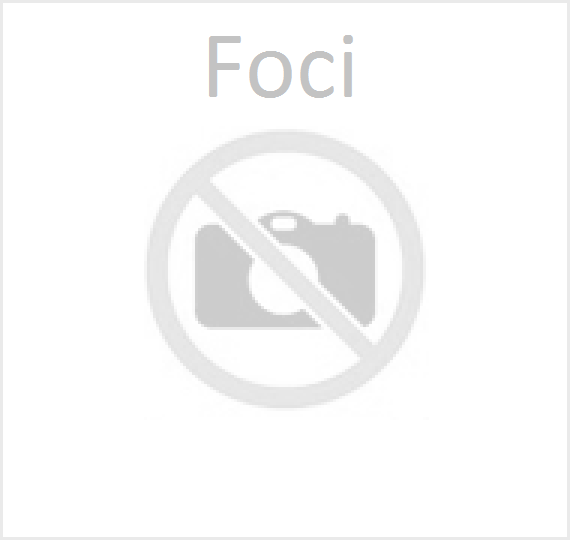

Supplement: Supplementary file 2 — GUI FoCo with source code. (ZIP 69 kb) [file 12859_2015_816_MOESM2_ESM.zip › Additional file 2/NoFociImage.png]

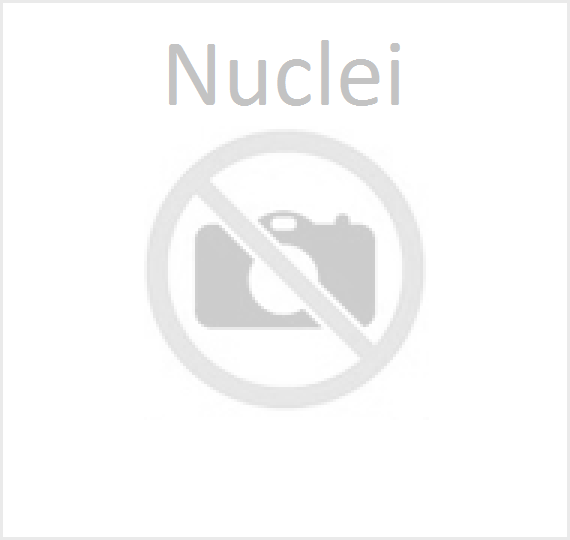

Supplement: Supplementary file 2 — GUI FoCo with source code. (ZIP 69 kb) [file 12859_2015_816_MOESM2_ESM.zip › Additional file 2/NoNucleiImage.png]

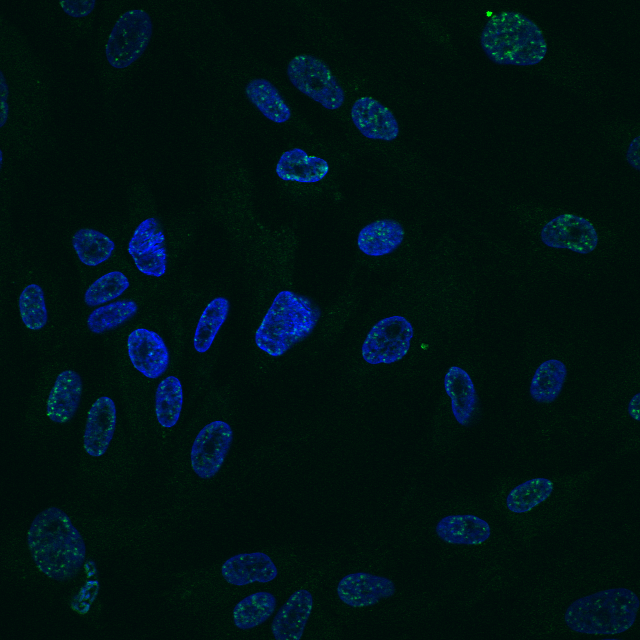

Supplement: Supplementary file 5 — Test image set. The test image set contains RGB and grayscale microscopy images of MRC-5 cells 1 hour after 2.5 Gy and 3 hours after 10 Gy irradiation and of non-irradiated MRC-5 cells. The included image of MRC-5 cells after 10 Gy irradiation (‘10Gy.tif’) was used for creating documentation for using FoCo. The user may use this image to verify if he/she is using the program correctly. (ZIP 1961 kb) [file 12859_2015_816_MOESM5_ESM.zip › Additional file 5/10 Gy/10Gy.tif]

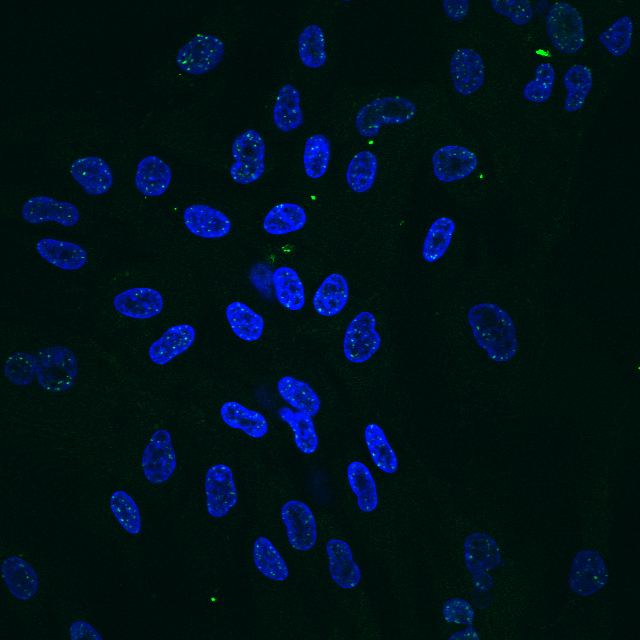

Supplement: Supplementary file 5 — Test image set. The test image set contains RGB and grayscale microscopy images of MRC-5 cells 1 hour after 2.5 Gy and 3 hours after 10 Gy irradiation and of non-irradiated MRC-5 cells. The included image of MRC-5 cells after 10 Gy irradiation (‘10Gy.tif’) was used for creating documentation for using FoCo. The user may use this image to verify if he/she is using the program correctly. (ZIP 1961 kb) [file 12859_2015_816_MOESM5_ESM.zip › Additional file 5/2.5 Gy/2Gy.tif]

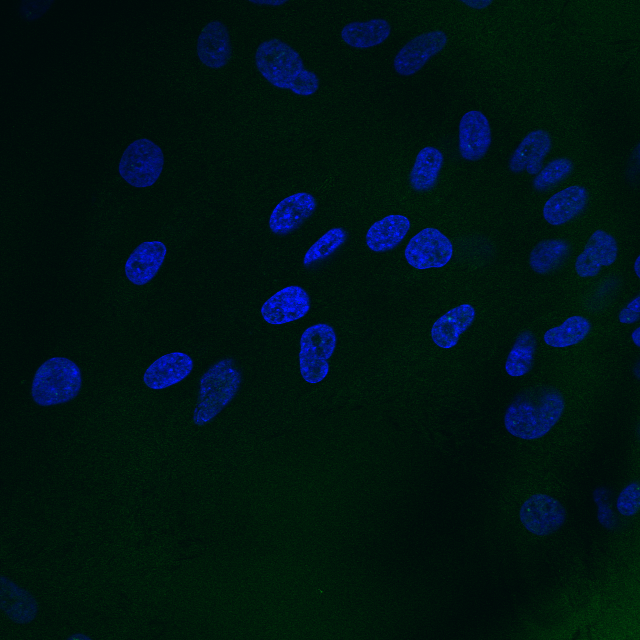

Supplement: Supplementary file 5 — Test image set. The test image set contains RGB and grayscale microscopy images of MRC-5 cells 1 hour after 2.5 Gy and 3 hours after 10 Gy irradiation and of non-irradiated MRC-5 cells. The included image of MRC-5 cells after 10 Gy irradiation (‘10Gy.tif’) was used for creating documentation for using FoCo. The user may use this image to verify if he/she is using the program correctly. (ZIP 1961 kb) [file 12859_2015_816_MOESM5_ESM.zip › Additional file 5/Control/Control.tif]

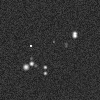

Supplement: Supplementary file 8 — Simulated foci images. We included simulated foci images from Fig. 4c–d used as representative images for demonstrating automatic foci quantifications. (ZIP 23 kb) [file 12859_2015_816_MOESM8_ESM.zip › Additional file 8/ControlFociImage.tif]

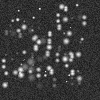

Supplement: Supplementary file 8 — Simulated foci images. We included simulated foci images from Fig. 4c–d used as representative images for demonstrating automatic foci quantifications. (ZIP 23 kb) [file 12859_2015_816_MOESM8_ESM.zip › Additional file 8/IrradiatedFociImage.tif]
