# Supplementary material for: FoCo: a simple and robust quantification algorithm of nuclear foci
Source: BMC Bioinformatics. 2015 Nov 21;16:392. doi: 10.1186/s12859-015-0816-5 (PMC4654864; doi:10.1186/s12859-015-0816-5)
Supplement: Supplementary file 3 — Documentation for using FoCo. (PDF 5946 kb) [file 12859_2015_816_MOESM3_ESM.pdf]

Simple and robust automatic quantification of γH2AX foci

Documentation for using FoCo

Anastasiya Lapytsko[1], Gabriel Kollarovic[1,2], Lyubomira Ivanova[1] , Maja Studencka[1], Jörg Schaber[1*]

[1] Institute for Experimental Internal Medicine, Medical Faculty, Otto von Guericke University, Pfälzer Platz 2, 39106, Magdeburg, Germany

[2] Cancer Research Institute, Slovak Academy of Sciences, Vlarska 7, 83391, Bratislava, Slovakia

[*] To whom correspondence should be addressed:

Jörg Schaber
Phone: +49 391 67 50227
Fax: +49 391 67 13312
Email: schaber@med.ovgu.de

1. Before starting FoCo

Before starting to work with FoCo the user needs to install Matlab with Image Processing Toolbox, ImageJ and Java Virtual Machine.

Additionally, the user needs MIJ that is a Java package for bi-directional communication and data exchange between Matlab and ImageJ. The user should download mij.jar and ij.jar from the web page http://bigwww.epfl.ch/sage/soft/mij/ and place it into the java directory of Matlab, e.g., for Windows Machine 'C:\Program Files\MATLAB\R2008b\java\'. This web page also contains additional information about MIJ.

2. Starting FoCo

FoCo is a graphical user interface (GUI) created in graphical user interface design environment (GUIDE) of Matlab. Before running FoCo the user should start Matlab. In Matlab the user should set the path to the folder with FoCo files as a current directory and start GUIDE by entering ‘gui’ in Command Window of Matlab. These steps are represented in Figure 1.

The main FoCo window must appear and FoCo is ready to use (see Figure 2).

The main FoCo window is divided on three zones:

- left zone to upload an RGB image or two grayscale images and adjust parameters needed for the algorithm step by step;
- middle zone to preview nuclei and foci images uploaded for analysis;
- right zone to upload and analyze an image array using adjusted parameters from the left zone, display and save quantification results.

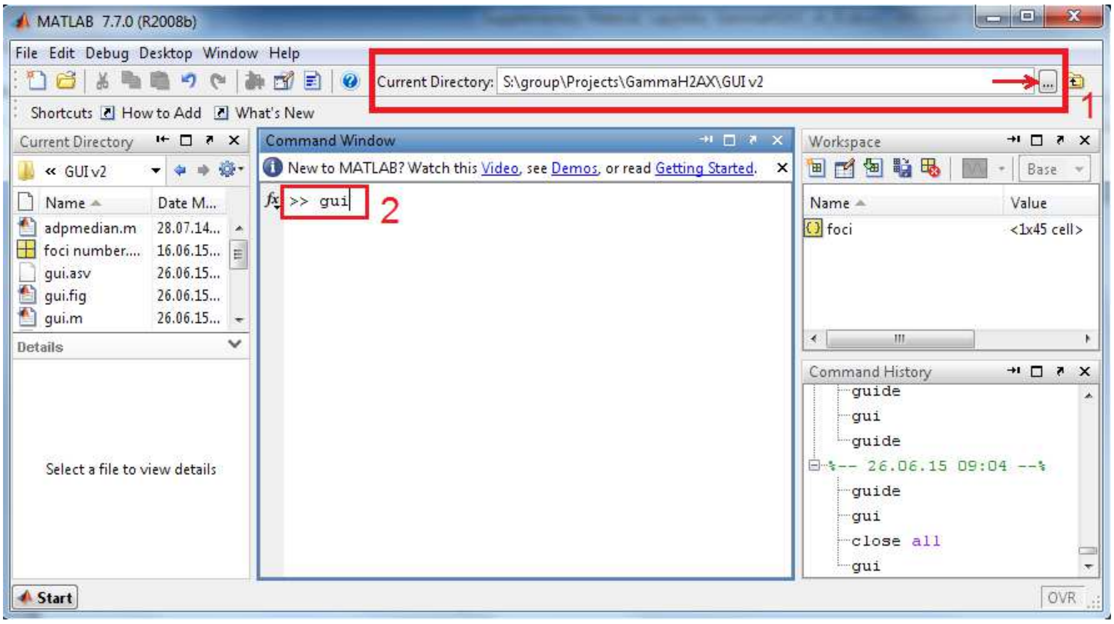

Figure 1. The scheme how to run FoCo in Matlab.

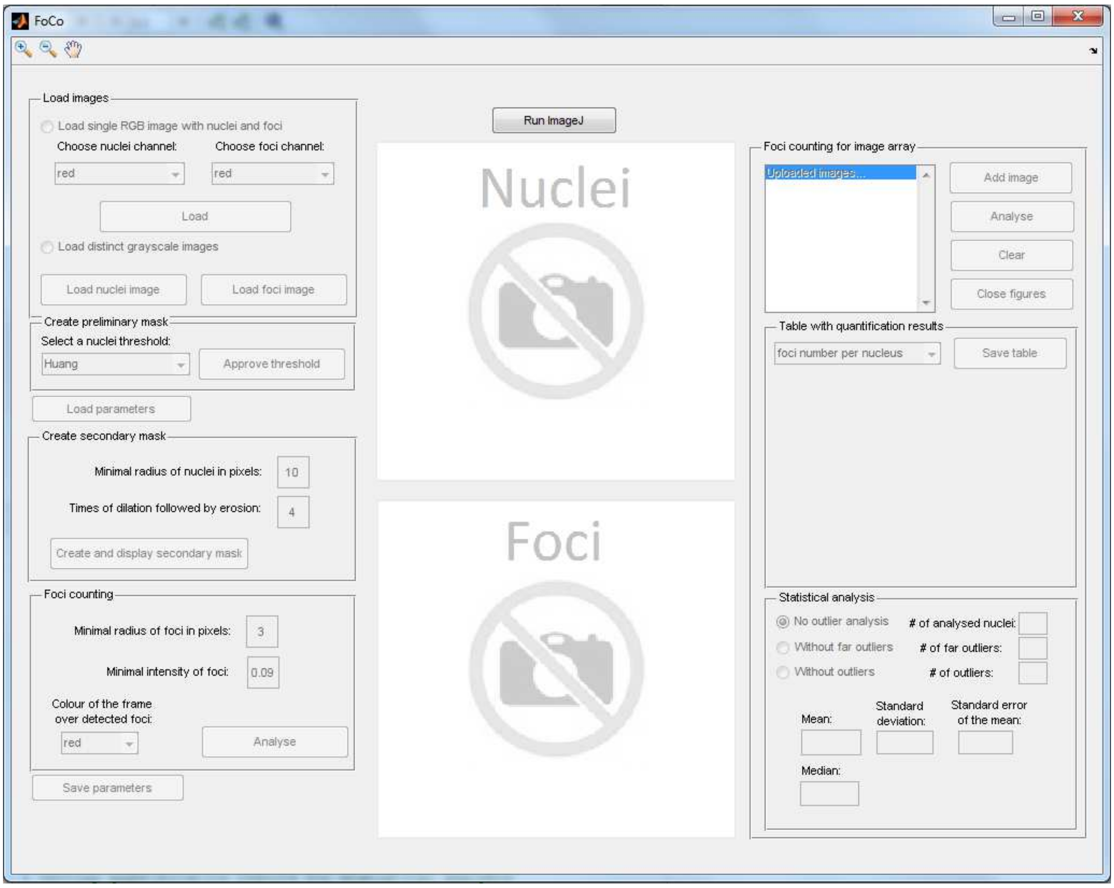

Figure 2. FoCo is started.

3. Run ImageJ from Matlab

Now the user should establish the connection between Matlab and ImageJ. For this the user should press the button 'Run ImageJ' and set the directory to the 'java' folder inside of the folder with Matlab program files, where MIJ files are located (see Figure 3). If the connection is established correctly, ImageJ will be started and the command window of Matlab will contain the connection description (see Figure 4).

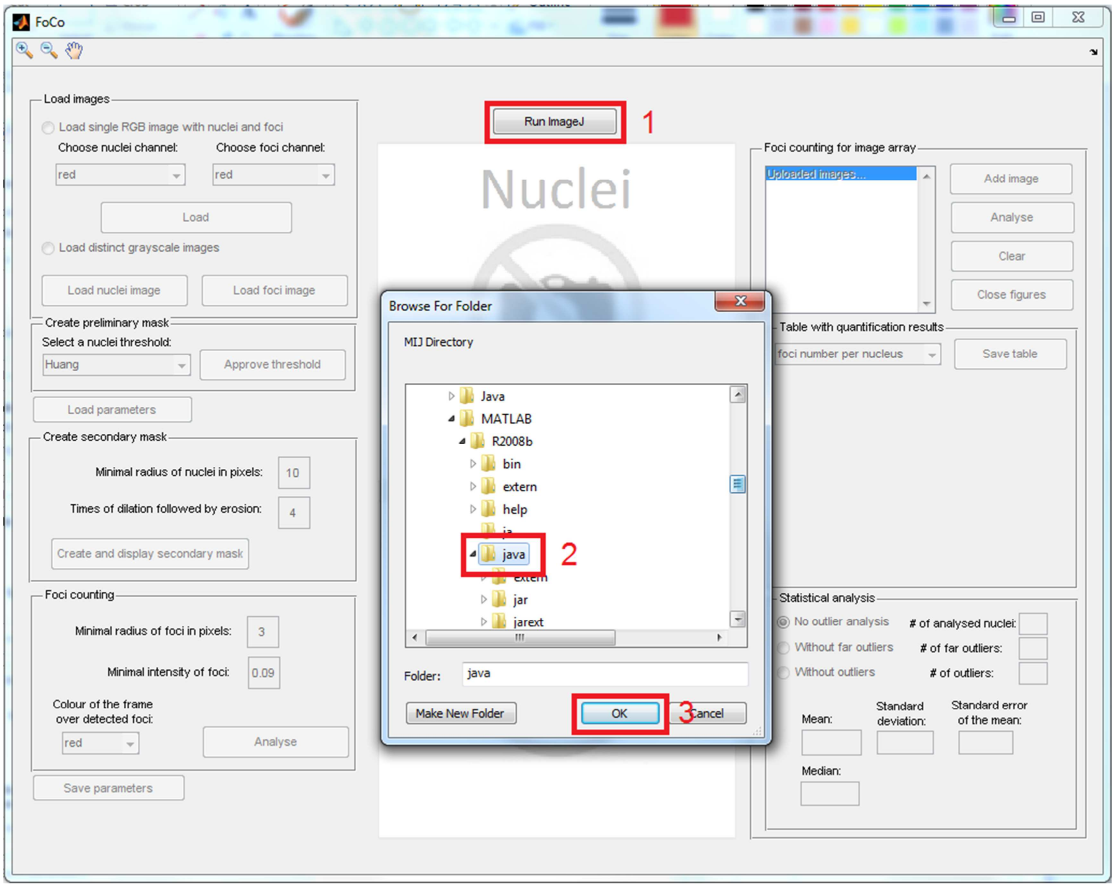

Figure 3. The first step of using FoCo is to run ImageJ and establish the connection between Matlab and ImageJ.

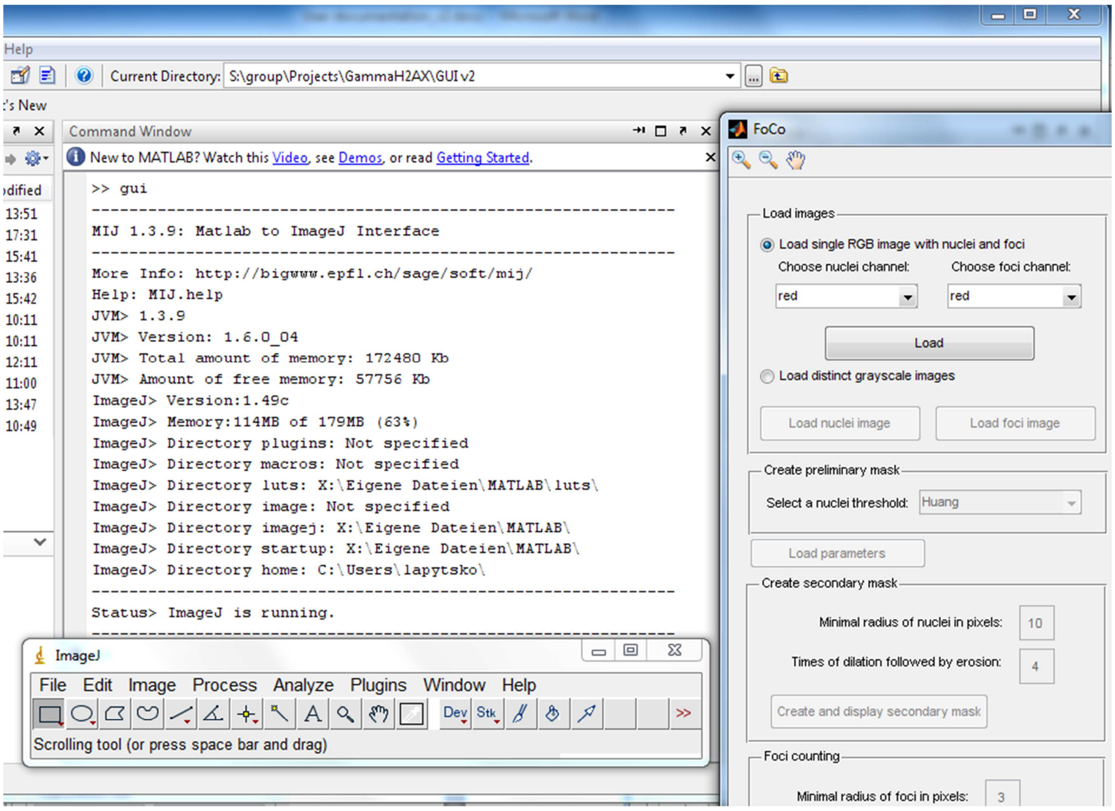

Figure 4. As the result of successfully established connection between Matlab and ImageJ, ImageJ is started and the command window of Matlab contains information about the connection.

4. Loading images for parameter adjustment

4.1. Loading foci image

After the connection between Matlab and ImageJ is established, the user should upload either a single RGB image that contains both nuclei and foci or two grayscale images with nuclei and foci, respectively. This image (images) will be further used for parameter adjustment.

In the case of the single RGB image the user should first select colors, which correspond to nuclei and foci channels: red, green or blue. Then the user should press the button ‘Load’ and select an image for analysis. The image will be automatically split on chosen channels and transformed into separate grayscale nuclei and foci images.

In the case of two grayscale images the user should press the button ‘Load nuclei image’ for uploading a grayscale image with nuclei and the button ‘Load foci image’ for uploading the corresponding grayscale foci image.

Successfully uploaded nuclei and foci images will be displayed in the middle zone of FoCo (see Figure 5).

Here, as an example we uploaded and analyzed the RGB image ‘10Gy.tif’ containing both nuclei and foci. The image is located in the subfolder ’10 Gy’ of the folder ‘Images for testing’ from the *sourceforge*. The user may upload this image and use to verify if he/she is using the program correctly.

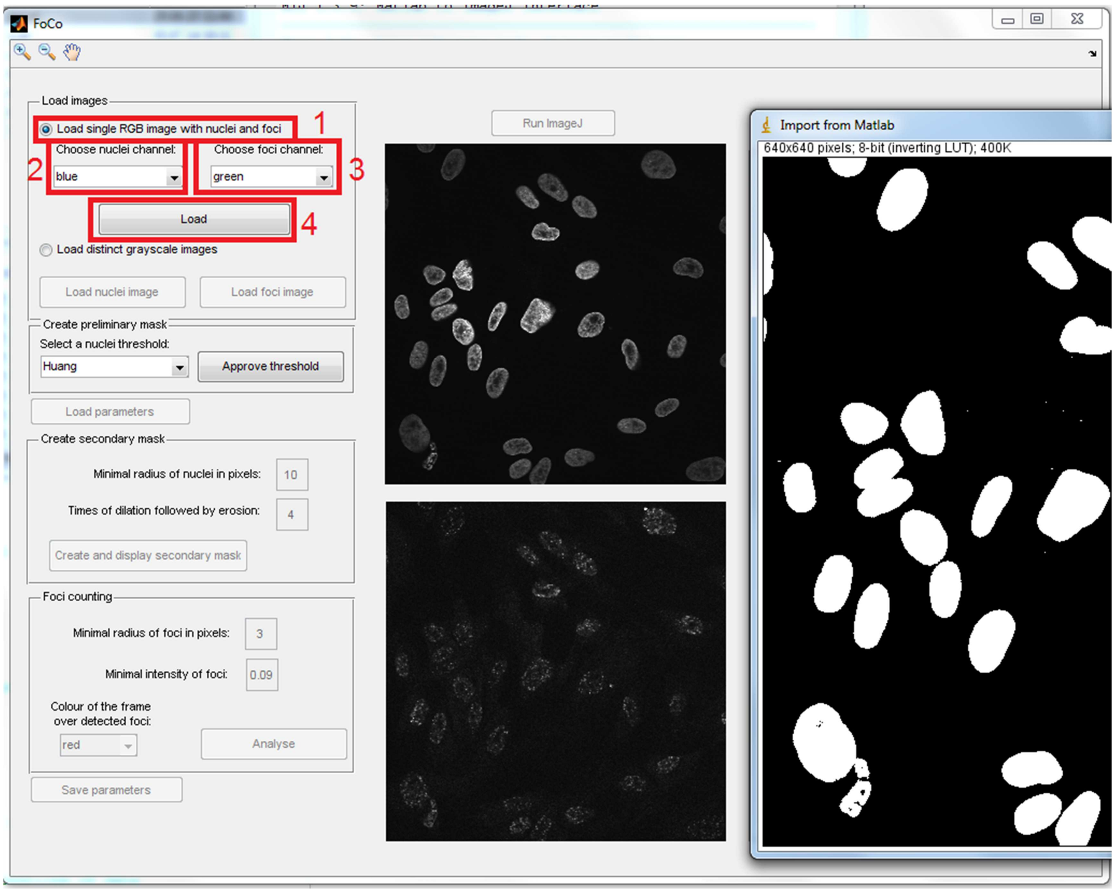

Figure 5. Loading a single RGB image.

4.2. Creating a preliminary nuclei mask

The next step is creating a preliminary nuclei mask for the nuclei image. After the user uploads the image for analysis, FoCo thresholds the nuclei component by ‘Huang’ method and displays the result in the separate window called ‘Import from Matlab’ (see Figure 5).

If the user is satisfied with thresholding results, the user should press the button ‘Approve threshold’. Otherwise the user should select another method for thresholding nuclei image by clicking the pop-up menu ‘Select a nuclei threshold’ (see Figure 6). The ‘Default’ method corresponds to modified

IsoData algorithm. The result of thresholding by selected method will be displayed in the separate window called ‘Import from Matlab’. After getting the satisfactory result the user should press the button ‘Approve threshold’.

Note that the user may upload parameter values including thresholding method, which were used and saved in one of previous sessions, by pressing the button ‘Load parameters’.

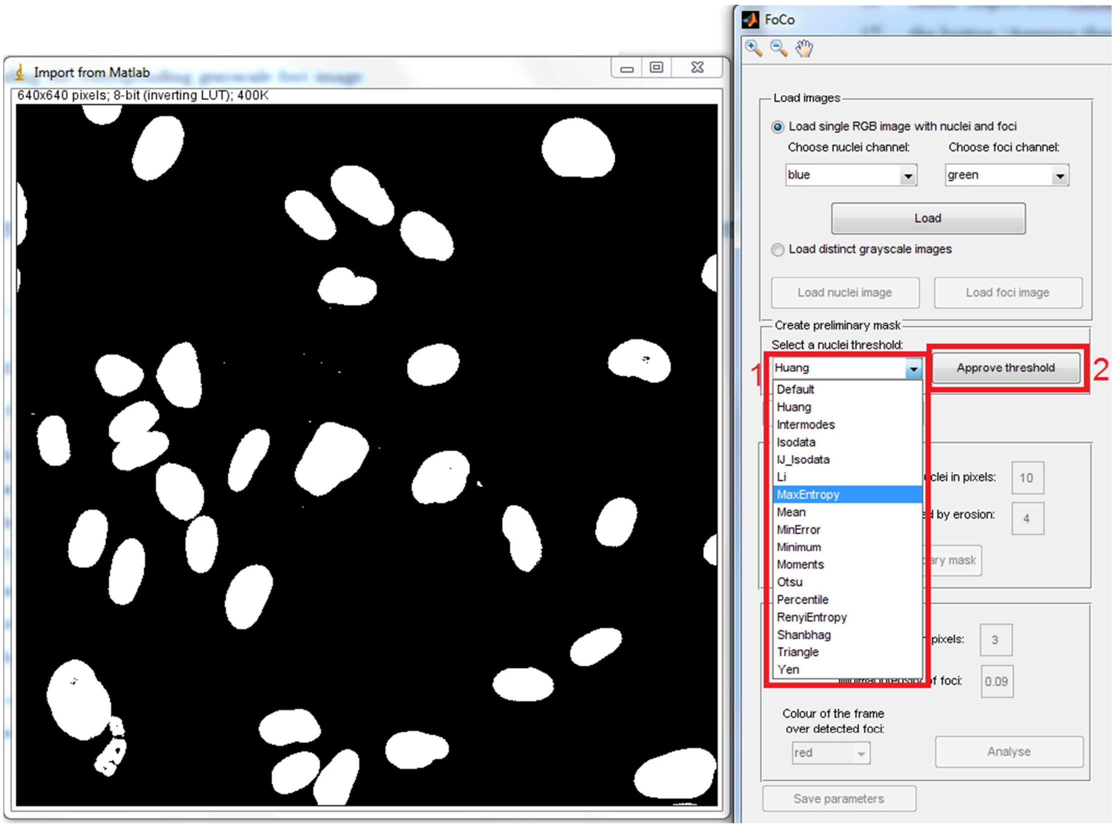

Figure 6. Selecting a threshold method for creating a preliminary mask of the nuclei image.

4.3. Creating a secondary nuclei mask

For creating a secondary nuclei mask, the user should input two parameters:

- ‘Minimal radius of nuclei’ in pixels with integer value greater than 0. This parameter is used to remove mask elements that do not represent nuclei (dust elements, etc.) and have radius less than average nuclei have.
- ‘Times of dilation followed by erosion’ with integer value greater than 1. This parameter means how many times elements of the mask will be dilated and subsequently eroded. In such a way we restore mask elements that have “c-shape” and that do not cover nuclei completely.

After the user presses the button ‘Create and display secondary mask’, the resulting mask appears in FoCo at the bottom of the middle zone (see Figure 7).

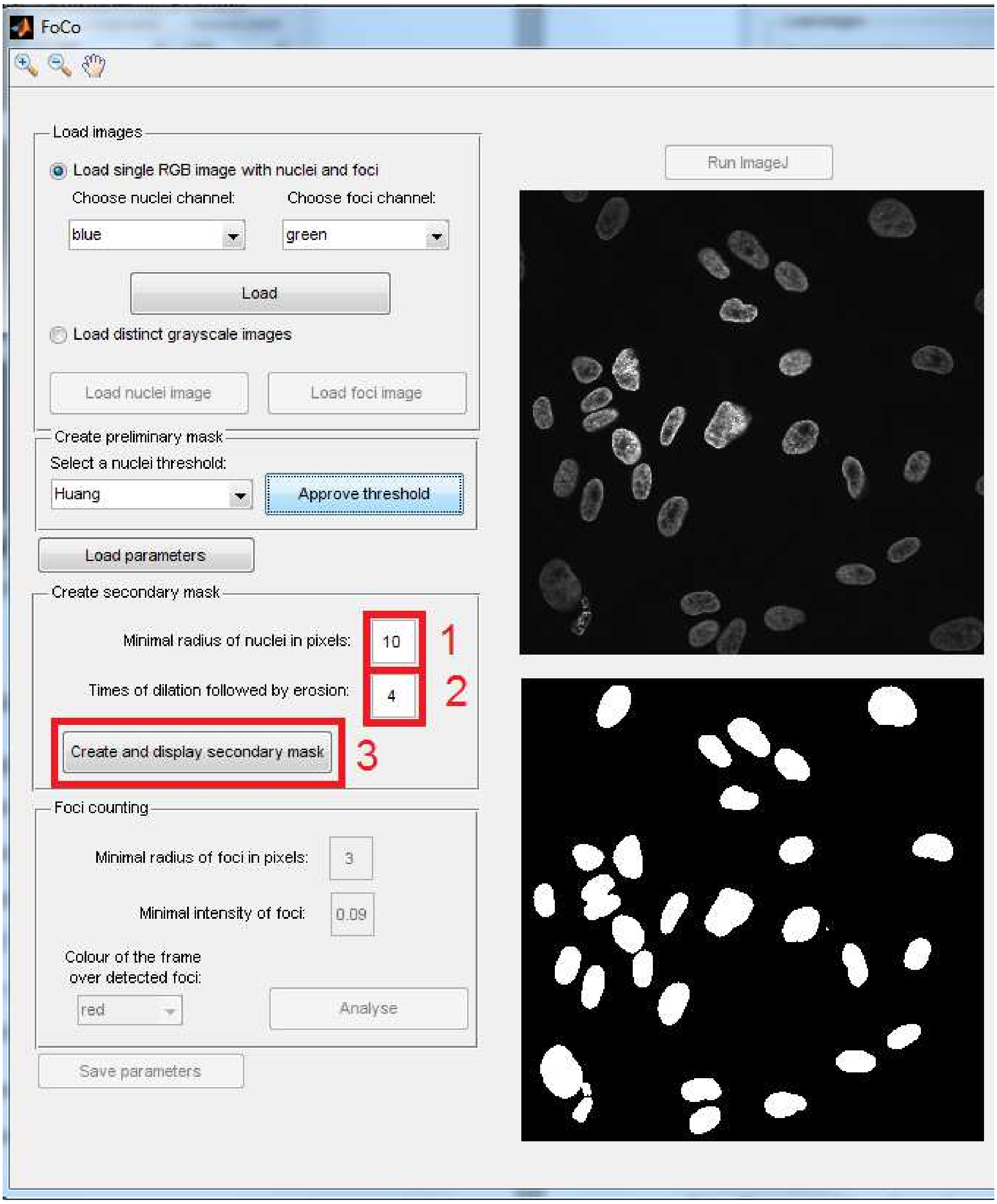

Figure 7. Creating the secondary nuclei mask.

4.4. Count foci

For counting foci the user should input two parameters:

- ‘Minimal radius of foci’ in pixels with integer value greater than 0. This parameter is used for estimating and subtracting background of the foci image.
- ‘The minimal intensity of foci’ with real value between 0 and 1. This parameter is used to exclude potential foci or their parts if their intensity is under certain level.

For optimizing values of these parameters we recommend to follow the algorithm represented in sections S3, S4 of Additional file 1 from our paper.

Additionally, the user should select the color of frames, which FoCo will put over detected foci. Borders of detected nuclei will be designated by white lines.

Finally, the user should press the button ‘Analyse’ (see Figure 8). FoCo processes the image and adds the analyzed image to the image array in the right zone (see Figure9). In the case of analyzing two grayscale images the formed image will be added with the name of the nuclei image. If the user clicks the name of the image at the array, nuclei and foci components of it will be displayed at the top and at the bottom of the middle zone, respectively (see Figure 9). Additionally, the original image with detected nuclei and foci will appear in the separate figure (see Figure 10). For closing all open figures the user should press the button ‘Close figures’.

If the user uploaded for analysis two grayscale images, then FoCo forms from them a single image, which contains both nuclei and foci designated by blue and green colours, respectively, and marks detected nuclei and foci on it.

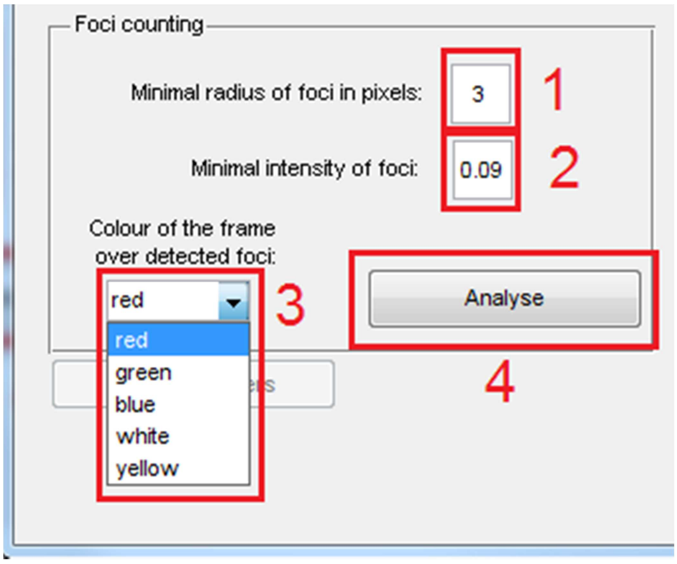

Figure 8. Counting foci.

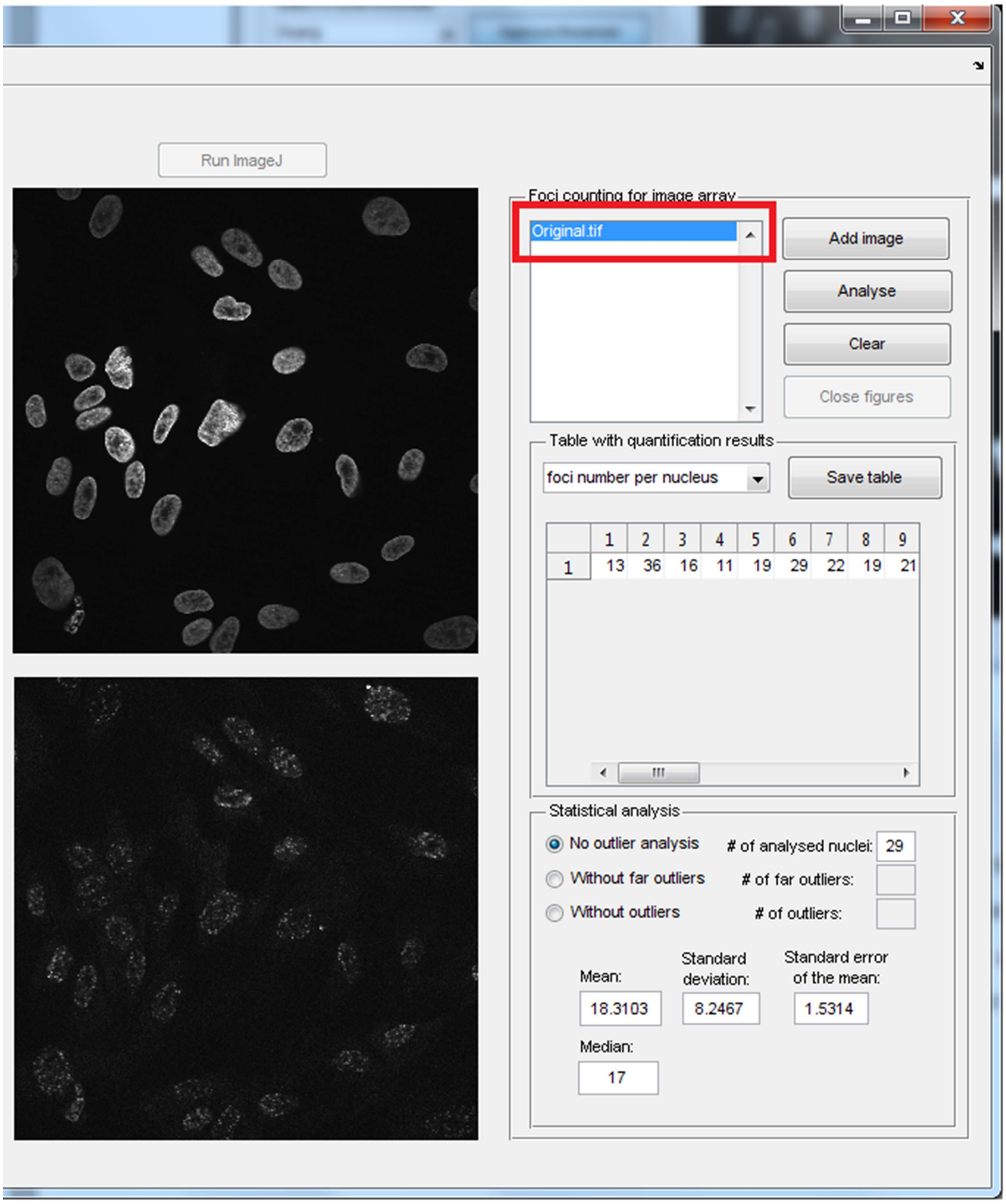

Figure 9. Adding the analyzed image to the image array and displaying quantification results.

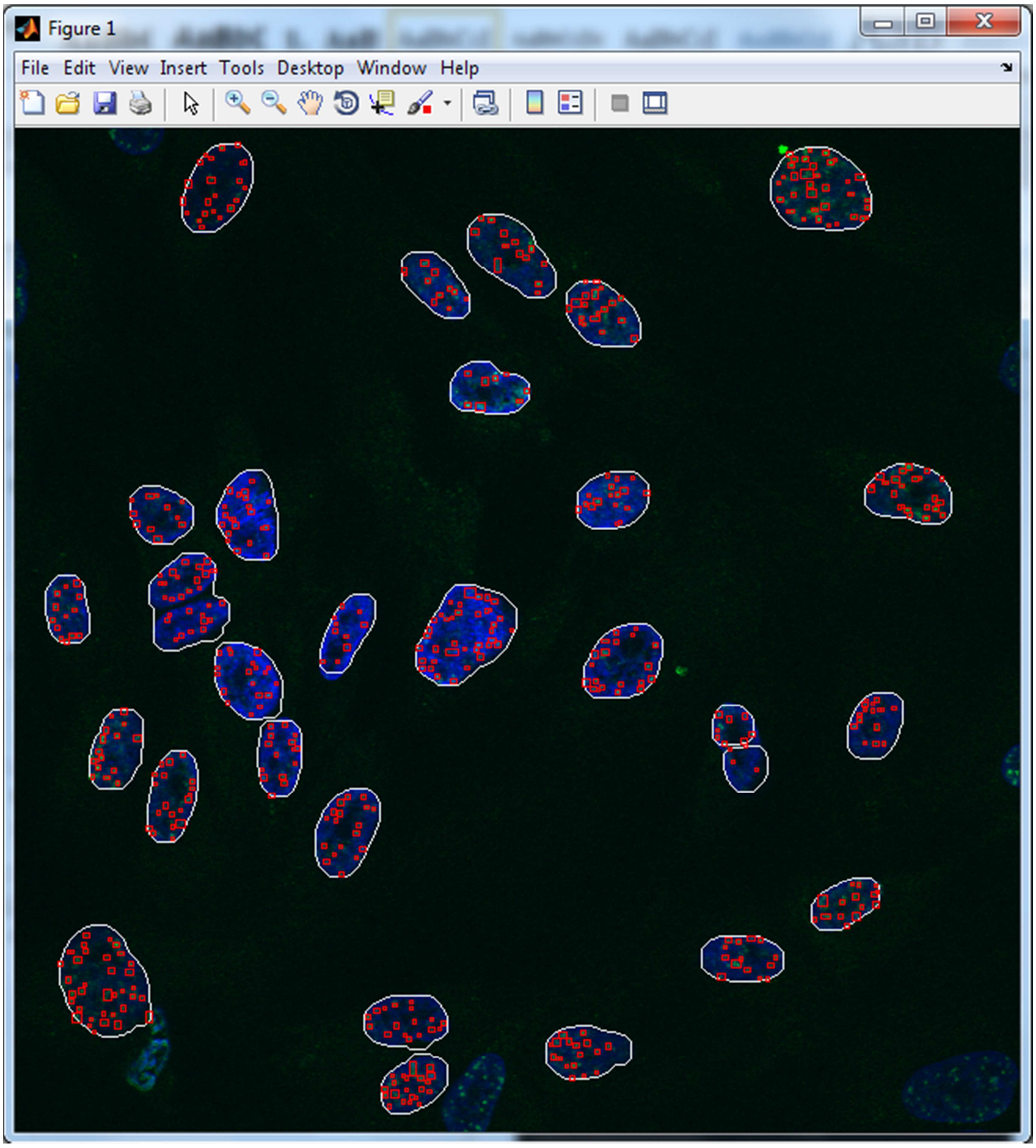

Figure 10. The original RGB image with detected nuclei (white lines) and foci (red frames).

For saving parameter values used for nuclei and foci detection the user should press the button 'Save parameters'.

5. Analysis of the image array and displaying quantification results

In order to analyze more images using parameters, which were adjusted in the left zone of FoCo, the user should first add images to be processed to the image array. For this the user should press the button 'Add image', select one

or several images to be processed and press 'Open' (see Figure 11). Added images will appear in the image array (see Figure 12).

For counting foci on images from the image array the user should press the button 'Analyse' (see Figure 12).

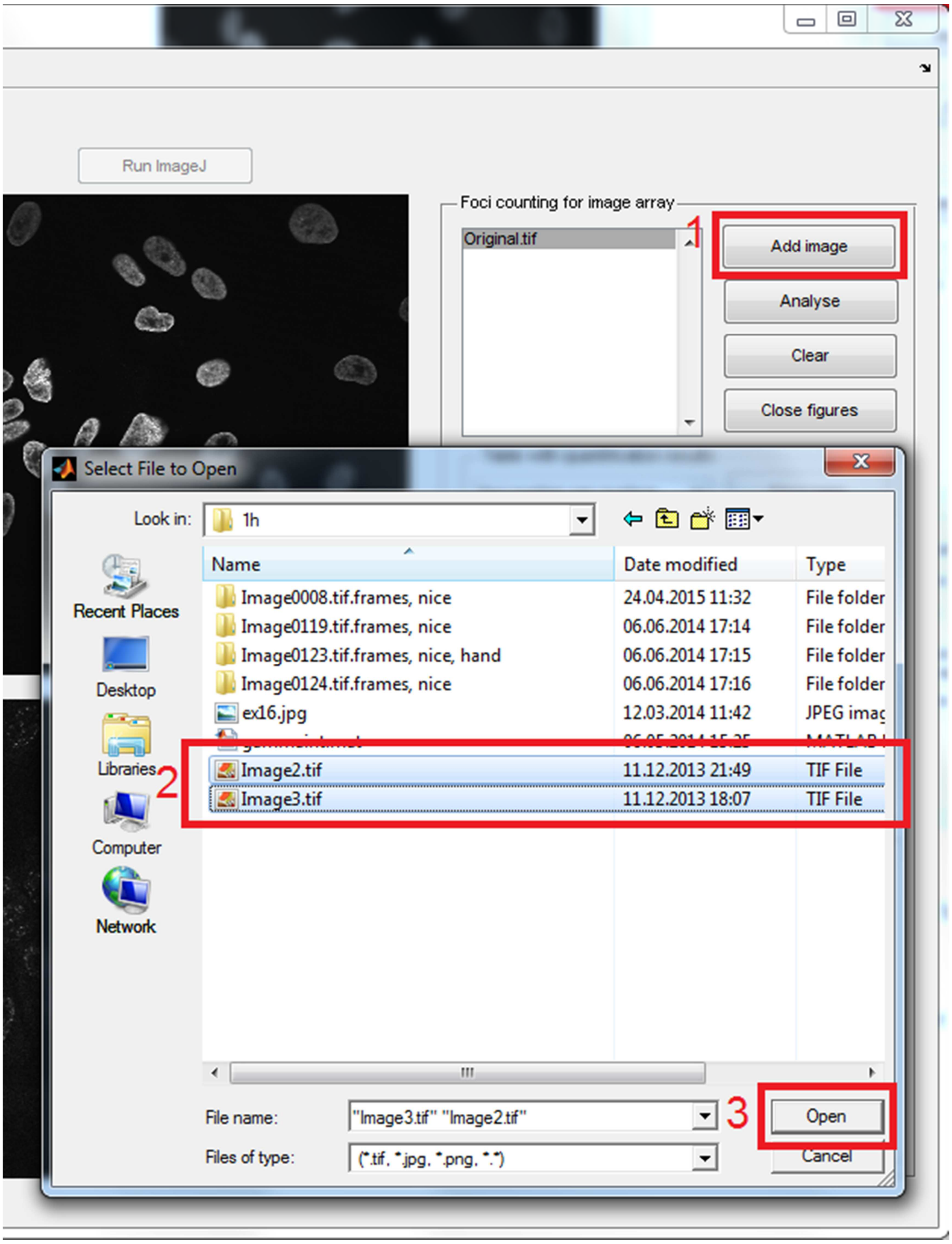

Figure 11. Adding images to the image array.

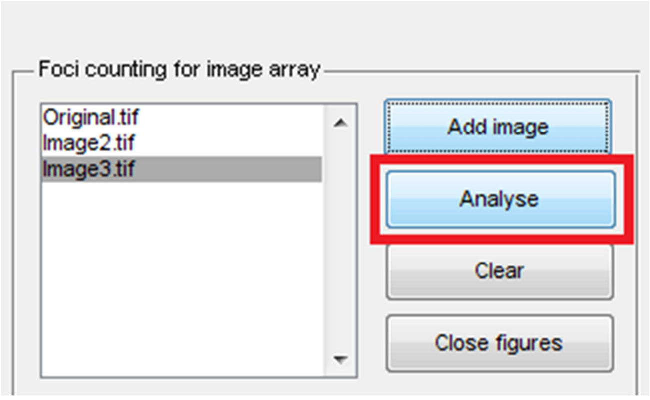

Figure 12. The analysis of the image array consisting of several images. For analysis FoCo uses parameters adjusted at the left zone.

For clearing the image array and deleting all quantification results the user should press the button 'Clear'.

Additionally, the right zone contains a table with quantification results. By clicking at the pop-up menu, the user may select, which information about analyzed images should be displayed in the table: foci number per nucleus or a mean γH2AX-associated fluorescence intensity within cell nuclei (see Figure 13). Rows of the table correspond to images at the array. Columns correspond to internal unique numbers of the recognized nuclei of the image. Thus, each cell with position (i, j) contains the information for the nucleus j of the image i from the array. If the user clicks the cell (i, j), the image i from the array will appear in the separate figure with a white frame over the nucleus j (see Figure 14).

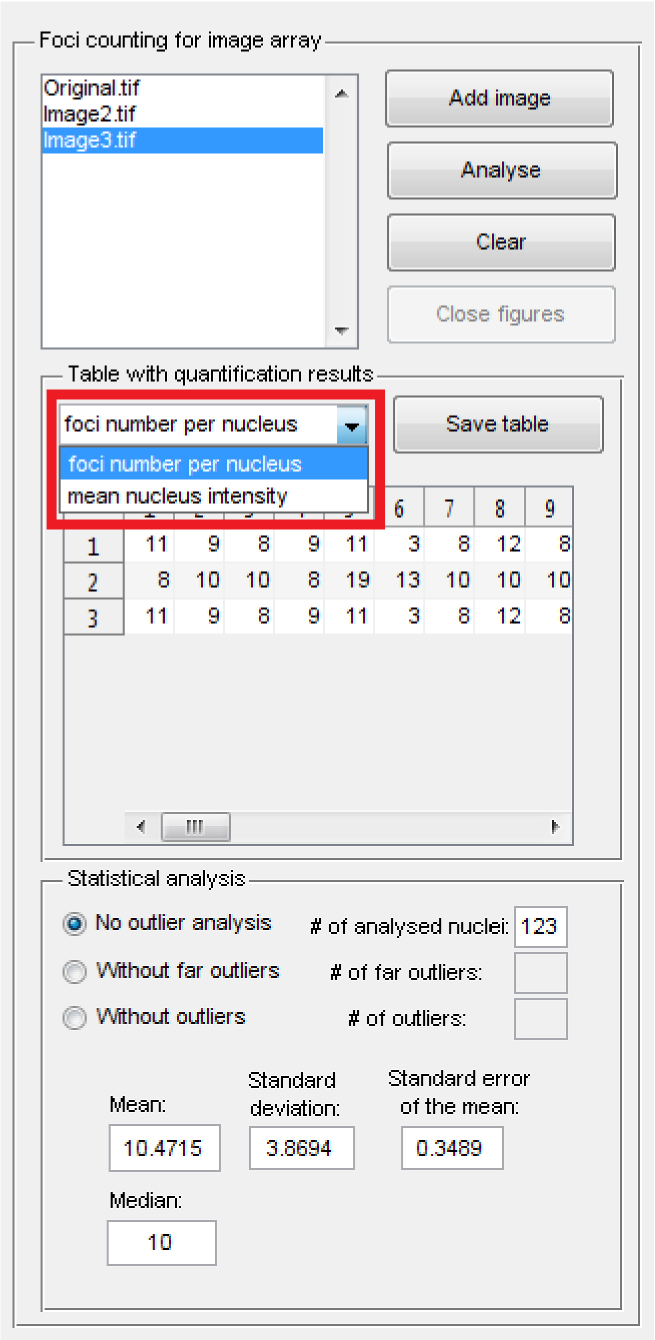

Figure 13. The table with quantification results.

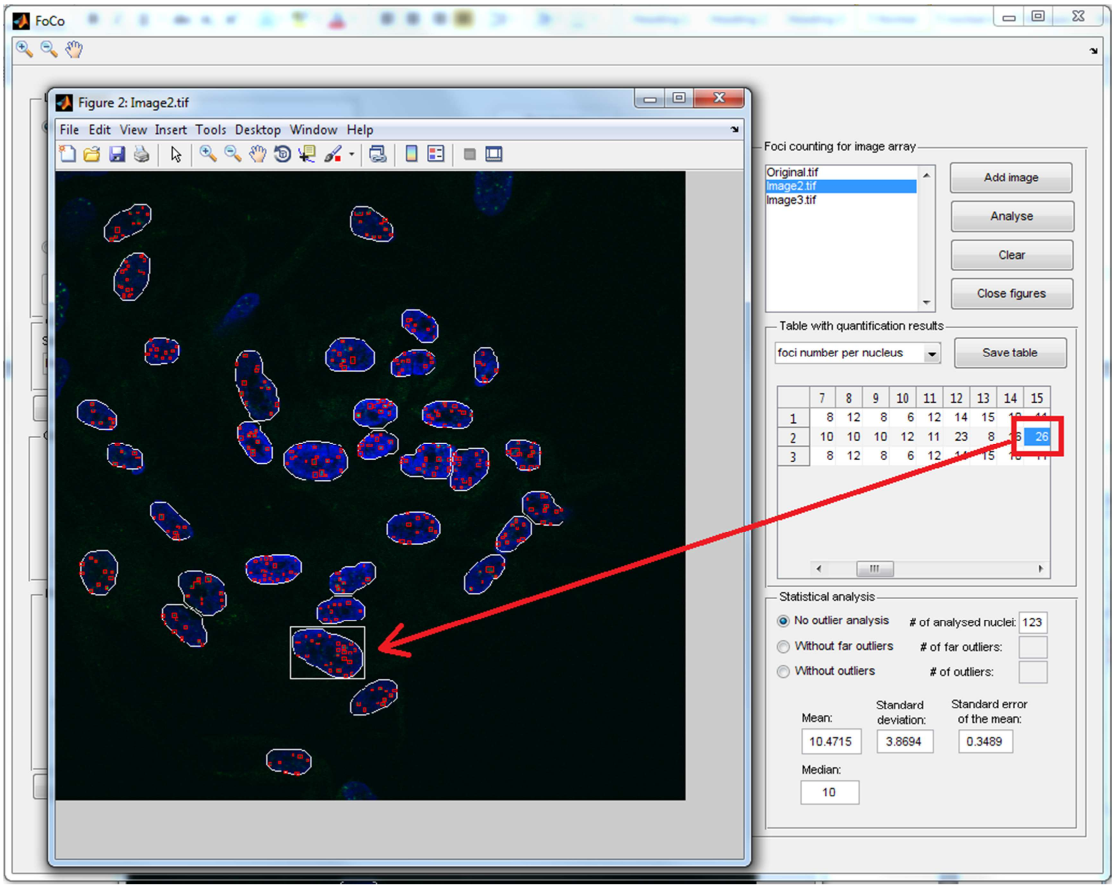

Figure 14. Connecting quantification results to the corresponding nucleus.

Note that the table with quantification results is also stored inside of the variable ‘foci’ in the Workspace of Matlab. For displaying quantification results in the Workspace the user should perform double click on ‘foci’ (see Figure 15). Then the user can select the data in the appeared table and paste them directly to Microsoft Excel for further processing and storing. Additionally, by pressing the button ‘Save table’ the user may save the displayed table with quantification results in ‘.mat’ format.

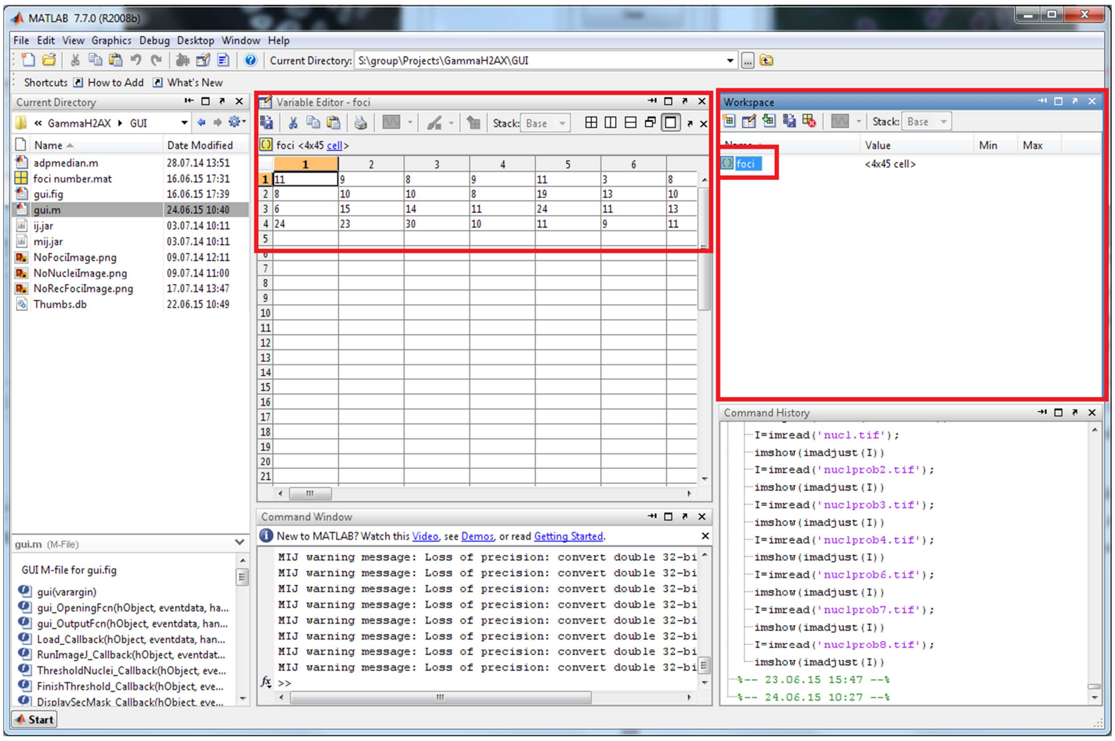

Figure 15. Quantification results will automatically appear in the workspace of Matlab inside of the variable ‘foci’ and can be used for further processing.

Finally, the bottom part of the right zone contains a basic statistical analysis of quantification results displayed in the table (see Figure 16): the mean value, the standard deviation, the standard error of the mean and the median value. Also it displays the number of analyzed nuclei.

In order to exclude far outliers or outliers the user should select the corresponding radio button ‘Without far outliers’ or ‘Without outliers’. Additionally, the number of detected far outliers or outliers is displayed. The procedure of outlier detection is described in the section S7 of Additional file 1 from our paper.

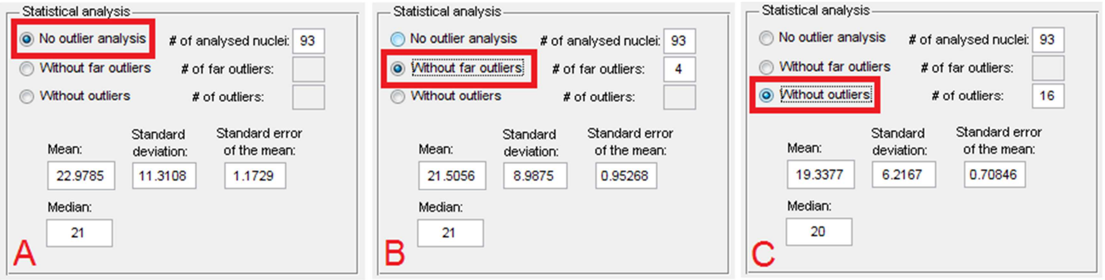

Figure 16. Statistical analysis of quantification results. **(A)** Analysis with outliers. **(B)** Without far outliers. **(C)** Without outliers.

6. Error: Java heap space

During the FoCo performance an error ‘Java heap space’ may occur (see Figure 17). It happens because of lack of memory for java virtual machine during image processing.

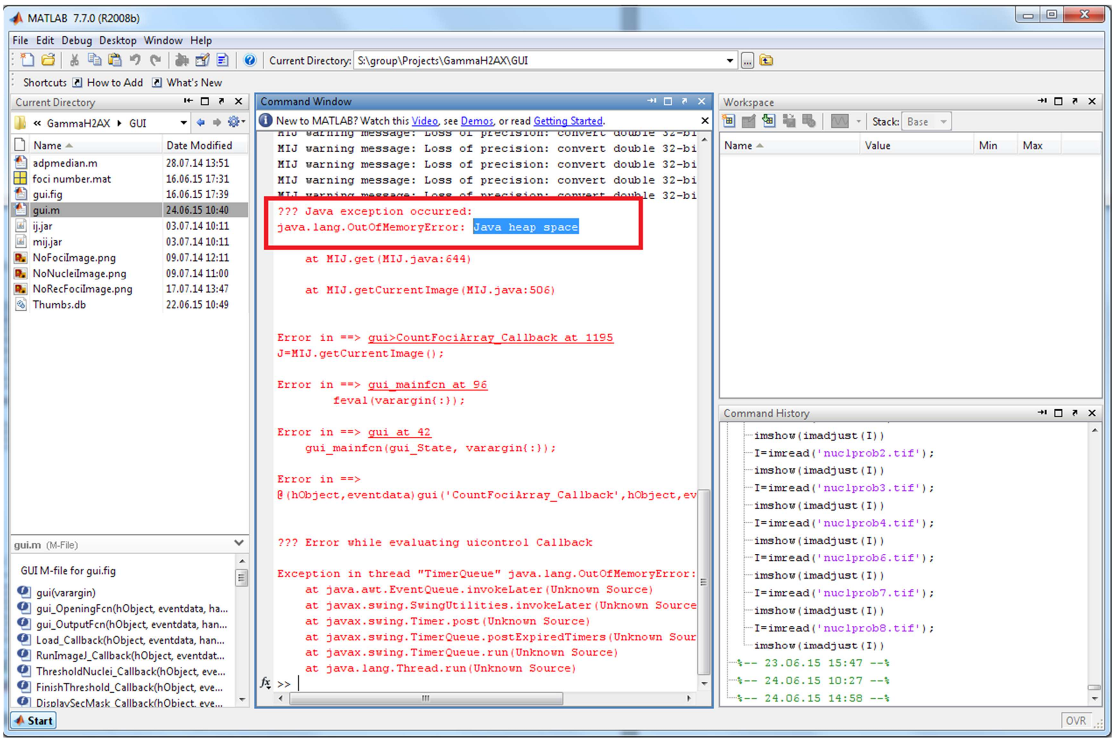

Figure 17. The error ‘Java heap space’ during the FoCo performance.

In order to solve this problem the user should increase the amount of memory for java virtual machine, e.g., by implementing commands described here:

http://www.mathworks.com/matlabcentral/answers/92813-how-do-i-increase-the-heap-space-for-the-java-vm-in-matlab-6-0-r12-and-later-versions

However, the error may occur again as long as java virtual machine is running out of memory. Therefore, if the error occurs, we advise to close all unnecessary displayed figures by pressing the button ‘Close figures’. This will release the memory for java virtual machine. Further, the user should restart processing of the image array by pressing the button ‘Analyse’ (see Figure 11).
